# *KRAS* mutated lung adenocarcinoma responds to pan-ERBB and Aurora kinase inhibitors

**DOI:** 10.1038/s41698-025-01242-8

**Published:** 2026-01-12

**Authors:** Iris Z. Uras, Marija V. Trkulja, Abdelrahman K.A.A. Salama, Jaqueline Horvath, Khushi Asnani, Christoph Trenk, Stefan Kubicek, Martin Bilban, Herwig P. Moll, Emilio Casanova

**Affiliations:** 1 Institute of Pharmacology, Center of Physiology and Pharmacology & Comprehensive Cancer Center (CCC), Medical University of Vienna, Vienna, Austria; 2 CeMM Research Center for Molecular Medicine of the Austrian Academy of Sciences, Vienna, Austria; 3 Department of Laboratory Medicine & Core Facilities, Medical University of Vienna, Vienna, Austria; 4 Ludwig Boltzmann Institute for Hematology and Oncology, Medical University of Vienna, Vienna, Austria

**Keywords:** Non-small-cell lung cancer, Targeted therapies

## Abstract

*KRAS* mutations are prevalent in lung adenocarcinoma (LUAD). Although KRAS-targeted therapies such as KRAS-G12C inhibitor sotorasib are now clinically available, their durability is limited by rapid resistance development, underscoring the need for novel strategies. Through high-throughput drug screening, we identified Aurora kinase (AURK) inhibitors as potent enhancers of afatinib efficacy in *KRAS* mutant LUAD models. ERBB/AURK co-inhibition synergized to suppress cell viability, clonogenicity, and tumor growth, mediated by induction of apoptosis, G_2_ → M cell cycle arrest, and disruption of compensatory signaling pathways. Mechanistically, dual inhibition activated pro-apoptotic programs, while impairing mitotic and survival pathways, as confirmed by phospho-proteomic and transcriptomic analyses. Notably, co-targeting ERBB and AURK effectively overcame resistance in afatinib- and sotorasib-refractory models, wherein bypass activation of EGFR, ERK, and AURK was observed. Given the limited survival benefit associated with KRAS-targeted therapies and rapid emergence of resistance in clinical settings, our findings establish ERBB/AURK co-inhibition as a promising therapeutic strategy to improve durability of response and combat acquired resistance in *KRAS* driven LUAD.

## Introduction

As global life expectancy increases, the burden of chronic diseases continues to rise, with cancer representing a leading cause of death worldwide. Among all malignancies, lung cancer remains the most common cause of cancer-related mortality, surpassing breast, colorectal, and prostate cancers combined^1^. Non-small cell lung cancer (NSCLC) accounts for approximately 85% of lung cancer cases, with lung adenocarcinoma (LUAD) representing the predominant histological subtype.

Activating mutations in the *KRAS* oncogene occur in over 30% of LUAD cases, with *KRAS*-G12C emerging as the most prevalent variant, comprising roughly half of all *KRAS* mutations. *KRAS* encodes a membrane-associated GTPase that transduces extracellular signals through critical oncogenic pathways such as MAPK/ERK and PI3K/AKT/mTOR. Mutations impairing GTP hydrolysis lock KRAS in an active GTP-bound state, driving sustained proliferative signaling. *KRAS* mutated LUADs are frequently associated with tobacco exposure, elevated PD-L1 expression, high tumor mutational burden, and an inflamed tumor microenvironment. These features are generally linked to improved responses to immune checkpoint inhibitors^2^. However, clinical outcomes remain heterogeneous, influenced by co-occurring genomic alterations in *STK11*, *KEAP1*, *TP53*, *SMARCA4*, and *CDKN2A/CDKN2B*^3–8^. These alterations can markedly shape tumor biology and modulate the immune landscape. Nonetheless, durable responses remain elusive, and no curative therapy currently exists for this molecular subset.

The development of small-molecule KRAS-G12C inhibitors, such as sotorasib and adagrasib^9,10^, marked a significant breakthrough in targeting a protein long considered “undruggable”. These compounds selectively bind the mutant cysteine in the switch II pocket of *KRAS*-G12C, trapping it in an inactive GDP-bound conformation^11^. Despite initial clinical success with response rates of 30-40% and accelerated regulatory approvals, drug resistance develops rapidly^12^. Notably, pivotal trials such as CodeBreaK 200 and KRYSTAL-12 fell short of the 6-month progression free survival (PFS) benchmark (5.6 months (sotorasib) vs 4.5 months (docetaxel); 5.5 months (adagrasib) vs 3.8 months (docetaxel)) that had seemed achievable during early phase trials^13–15^, raising concerns about the true therapeutic impact of anti-KRAS-G12C monotherapy.

Mechanisms of resistance are multifactorial, involving both genetic and non-genetic alterations. Mutations in the RAS/MAPK axis, epithelial-to-mesenchymal transition (EMT), and compensatory signaling via receptor tyrosine kinases (RTKs), including EGFR and other ERBB family members, have been implicated in both intrinsic and acquired resistance^12,16–24^. Several trials are thus exploring the benefit of KRAS-G12C inhibitors in combination with MEK/ERK inhibitors, PI3K inhibitors and SHP2 inhibitors, among others^25,26^. Recent studies further demonstrate that KRAS-G12C inhibition leads to heterogeneous cellular responses, with subsets of tumor cells escaping via rapid re-synthesis of active, drug-insensitive KRAS protein, maintained through EGFR and Aurora kinase (AURK) signaling^17,19^. These insights underscore the need for rational combination strategies that prevent or delay resistance and induce durable tumor regression.

In silico drug repurposing efforts have identified afatinib, a clinically approved, irreversible pan-ERBB (EGFR/ERBB2/ERBB4) tyrosine kinase inhibitor (TKI), as a candidate therapeutic against *KRAS*-G12C driven tumors^27^. This is supported by preclinical evidence of us and others showing that *KRAS* mutant LUADs require tonic input from activated ERBB family members for tumor initiation and progression^28–30^. While EGFR-selective TKIs initiate a rapid resistance due to induction and activation of non-EGFR ERBB members, afatinib suppresses compensatory ERBB signaling and effectively inhibits *KRAS* mutated LUAD models^28,29^. However, as with most targeted monotherapies, single-agent afatinib is unlikely to achieve durable remission.

To identify strategies that enhance the efficacy of ERBB inhibition in *KRAS* mutant LUAD, we performed a high-throughput chemical screen across *KRAS* mutant LUAD models. Among multiple candidates, AURK inhibitors emerged as potent enhancers of afatinib-induced growth suppression. Combination treatment with afatinib and pan-AURK inhibitor tozasertib synergistically impaired clonogenicity, induced robust apoptosis, and promoted cell cycle arrest through upregulation of pro-apoptotic (e.g., BIM, IFN-β) and cell cycle inhibitory (e.g., p21^WAF1/CIP1^) programs. Mechanistically, dual ERBB/AURK blockade disrupted adaptive resistance mechanisms, including feedback activation of EGFR, AURK, and TBK1 pathways. Importantly, this combination overcame resistance in models refractory to afatinib or the KRAS-G12C inhibitor sotorasib. These findings reveal ERBB and AURK co-targeting as a promising therapeutic strategy to improve outcomes in *KRAS*-driven LUAD, warranting further clinical investigation.

## Results

### Chemical screen reveals Aurora kinase inhibitors as enhancers of afatinib-induced growth inhibition in *KRAS* mutated lung adenocarcinoma

**Fig. 1:**
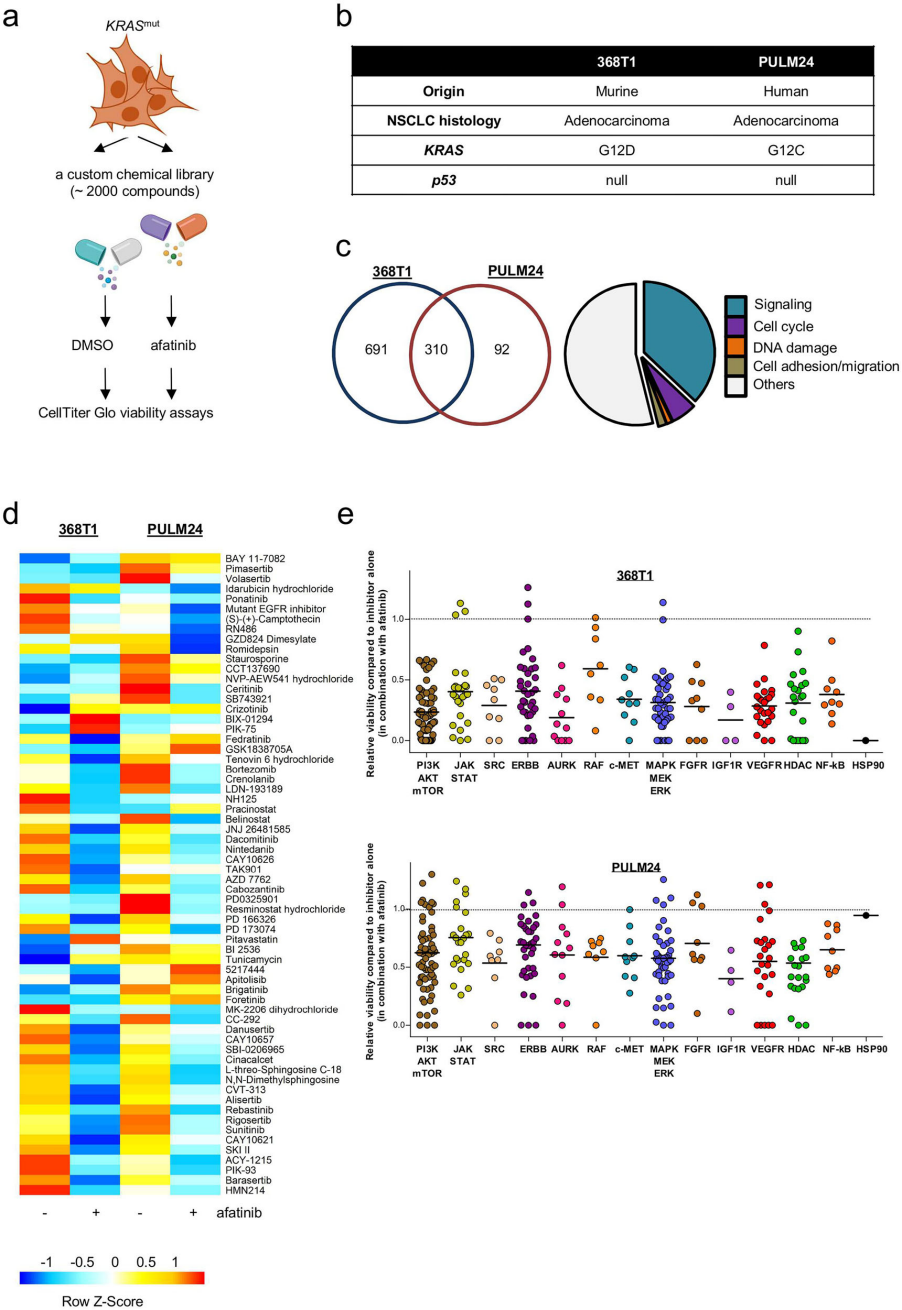
Focused chemical screen reveals hypersensitivity of *KRAS* mutated LUAD cell lines to several compounds when combined with afatinib. **a** A schematic of high-throughput screen to identify agents that enhance afatinib-induced growth inhibition. Cells were treated with each compound from the library (10–50 µM) in the absence and presence of afatinib (1 µM) (see Supplementary Fig. 1a). Cell viability was assessed by an ATP-dependent luminescent assay at 72 h. **b** Characteristics of NSCLC adenocarcinoma cell lines used in the drug screen. **c** A Venn diagram of agents that enhance afatinib-induced growth inhibition in 368T1 and PULM24 cells (see Supplementary Fig. 1b). 310 compounds were shared across both cell lines. 691 agents were unique for 368T1. 92 compounds were unique for PULM24. **d** Heat map shows compounds being effective in combination with afatinib in different cell lines tested. Blue, sensitivity; red, resistance. **e** An overview of growth inhibition by various pathway inhibitors plus afatinib. Viability by the combination of afatinib with each compound was normalized against that by each compound (set to 1) (see also Supplementary Fig. 2). Each circle represents one compound.

**Fig. 2:**
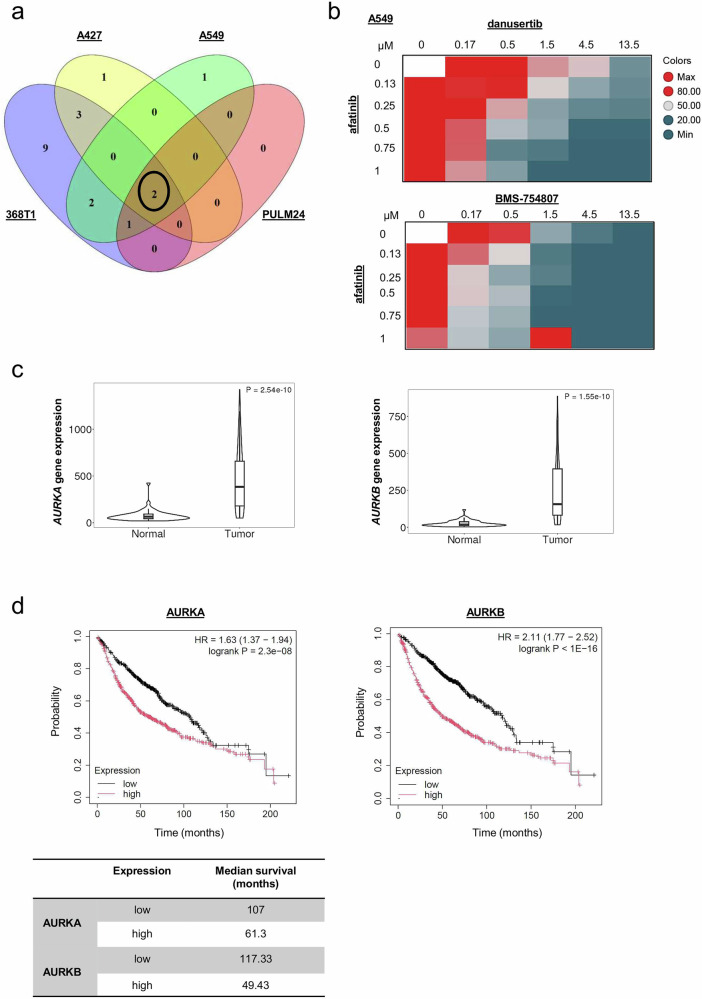
Combined ERBB and AURK inhibition reveals synergistic effects in *KRAS* mutated LUAD cell lines. **a** A Venn diagram of 25 agents combined with afatinib in 6 ×6 matrices. Two compounds are common across the four *KRAS* mutated LUAD cell lines (see also Supplementary Fig. 3). **b** Heat maps, depicting variation in drug interactions, are shown for afatinib combinations with the pan-AURK inhibitor danusertib (top) and the IGF1R inhibitor BMS-754807 in *KRAS* mutated A549 (bottom). Indicated compounds were tested with afatinib in 6×6 matrices. Each matrix included a dose-response of each drug alone together with solvent (DMSO) only; thus a 6×6 matrix included five doses each tested in combination (36 combinations in total). Following a 3 day incubation cell viability was measured by CellTiter-Glo (CTG) Viability Assay. Blue, dead; red, viable. **c** Relative mRNA expression of the indicated genes in paired lung adenocarcinoma and adjacent normal tissues using TNMplot.com^38,39^. Fold change mean (*AURKA*): 6.38; fold change mean (*AURKB*): 10.35. n = 57; **** *P* < 0.0001 (Mann-Whitney test). **d** High mRNA expression of *AURKA/B* in lung adenocarcinoma confers bad prognosis (KMplot.com)^38,39^. AURKA probe ID: 204092_s_at; AURKB probe ID: 209464_at; *n* (*AURKA* high) = 579; *n* (*AURKA* low)=582; *n* (*AURKB* high)= 576; *n* (*AURKB* low)=585; ****P < 0.0001 (Multivariate analysis).

To uncover combination strategies that enhance the efficacy of afatinib in *KRAS* mutant LUAD, we performed high-throughput drug screening using a curated library of ~2000 compounds, including FDA-approved agents, pathway-specific inhibitors, small-molecule epigenetic modulators, and natural products (Supplementary Fig. 1a)^31^. 368T1 and PULM24 cells expressing different *KRAS* mutations were treated with each compound, alone or in combination with afatinib, and relative viability inhibition was assessed (Fig. 1a, b). Approximately 300 overlapping hits were identified across both cell lines (Fig. 1c, d; Supplementary Fig. 1b). We focused on the efficacy of inhibitors targeting known resistance mechanisms of ERBB TKI and KRAS-G12C drugs. Inhibitors of AURK, PI3K/AKT/mTOR, JAK/STAT, ERBB, MET, MAPK/MEK/ERK, IGF1R, NF-κB, and HDAC further potentiated afatinib-induced growth suppression in both cell lines (Fig. 1e; Supplementary Fig. 2). Focused pairwise drug combination assays testing 25 prioritized compounds in combination with afatinib were performed across a panel of LUAD cell lines harboring various *KRAS* mutations (Fig. 2a; Supplementary Fig. 3). Synergy was determined by comparing the experimentally observed viability outcome of the combination to the predicted outcome of each single agent, using the statistical independence Bliss model^32^. Danusertib (pan-AURK inhibitor) and BMS-754807 (IGF1R inhibitor), both in phase II trials, demonstrated consistent synergy across all tested models (Fig. 2b; Supplementary Figs. 4, 5). Recent studies have underscored the therapeutic potential of IGF1R inhibition in LUAD. Combining IGF1R inhibitors with EGFR TKIs induces apoptosis in *EGFR* mutated tumors^33^. Furthermore, IGF1R inhibitors are especially effective in *KRAS* mutated LUAD, as these tumors rely on IGF1R kinase activity to activate the downstream PI3K pathway, promoting sustained proliferation^34^. Similar dependency has been observed in *KRAS* mutated pancreatic cancer, where IGF1/AKT signaling supports tumor cell survival and dormancy after oncogene ablation^35^. Inhibition of IGF1R in this context reduces residual disease burden and cancer recurrence, underscoring the broader relevance of IGF1 signaling in *KRAS* driven cancer.

Aurora kinases are key mitotic regulators whose inhibition causes segregation errors and cell death^36,37^. In lung cancer, high *AURK* expression predicts poor prognosis^38,39^ (Fig. 2c, d; Supplementary Fig. 6), and can enhance activation of EGFR/RAS effectors including AKT, ERK1/2, and EMT-associated proteins, promoting drug resistance and tumor invasion^40–43^. Conversely, oncogenic KRAS can upregulate AURK expression via transcriptional or post-transcriptional mechanisms^44^, suggesting a reciprocal feedback loop that reinforces oncogenic signaling. In *EGFR* driven LUAD, combined EGFR and AURKA/B inhibition is synergistic^40,43^, with clinical trials underway (NCT05017025, NCT04085315). Dual AURKA/B blockade selectively impairs *KRAS* mutant cell growth, with minimal impact on *KRAS* wild-type counterparts^44^. Furthermore, AURK inhibition is lethal in LUAD that has progressed on the KRAS*-*G12C inhibitors^45,46^. These findings motivated us to further investigate the therapeutic interaction between ERBB and AURK inhibition in *KRAS* driven LUAD.

### Co-inhibition of ERBB and Aurora kinase family members impairs cell clonogenicity and proliferation in *KRAS* mutant LUAD

**Fig. 3:**
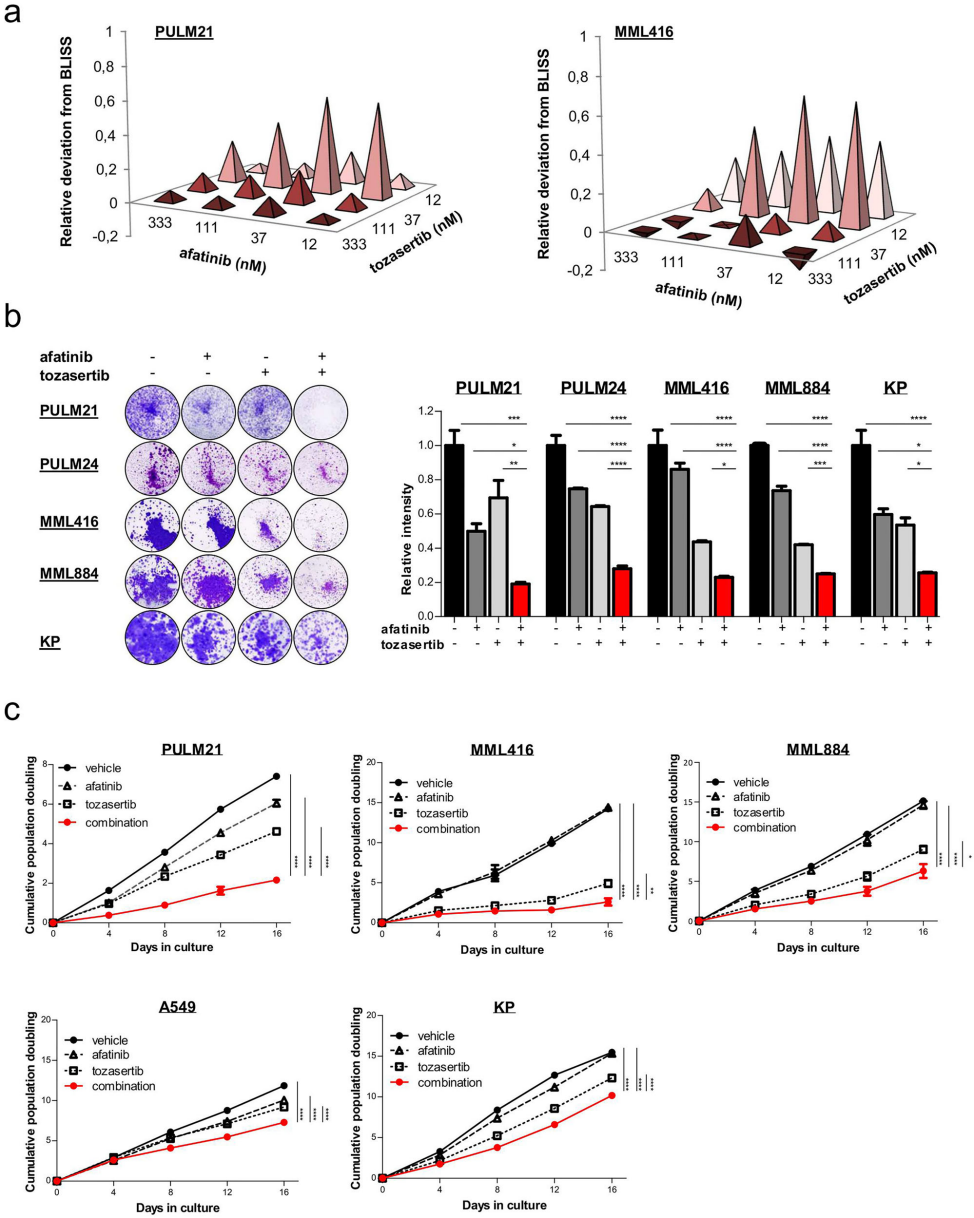
Combined afatinib and tozasertib reduce clonogenicity and growth of *KRAS* mutated LUAD cells. **a** Combined effect of afatinib with the pan-AURK inhibitor tozasertib exceeds Bliss prediction, indicating synergy at very low nanomolar concentrations. Analysis was carried out in triplicate. Values depicted represent absolute deviations. Observed values are divided through standard deviations (SDs) plus 15th percentile. Needle graphs indicate deviation from Bliss predicted synergy in *KRAS* mutated LUAD cells (PULM21 (human) and MML416 (murine)). **b** Crystal violet staining of the indicated *KRAS* mutated LUAD cell lines 7-10 days after treatment with DMSO or the indicated drugs. Human cell lines PULM21 and PULM24 were exposed to 37 nM of each compound. Murine cells (MML416, MML884 and KP) were exposed to 111 nM of each compound. The concentrations were based on the relative deviation from Bliss as shown in Fig. 3a. Representative images of crystal violet staining of colonies are shown (*n* = 3 per group). ImageJ was used for quantification. Data are presented as mean values ± standard error of the mean (SEM). *P < 0.05, **P < 0.01, ***P < 0.001, ****P < 0.0001 (One-way ANOVA with subsequent Bonferroni posttest). **c** Growth curves of the indicated cells treated with vehicle, single agent (PULM21, A549: 37 nM; MML416, MML884, KP: 111 nM), or the combination. Analysis was carried out in triplicate per cell line. Data are presented as mean values ± SEM. *P < 0.05, **P < 0.01, ****P < 0.0001, n.s. not significant (One-way ANOVA with subsequent Bonferroni posttest).

To confirm the specific role of Aurora kinases in afatinib sensitization, we performed combinatorial drug viability assays using afatinib and selective inhibitors targeting AURKA (alisertib, in phase III clinical trials), AURKB (BI-31266), or pan-AURKA/B/C (tozasertib, in phase II clinical trials) across different *KRAS* mutated LUAD lines (Supplementary Fig. 7a). Co-treatment of afatinib with selective AURK inhibitors exhibited pronounced synergy as validated by Bliss excess (Supplementary Fig. 7b), and significantly reduced cell viability at low nanomolar concentrations (Supplementary Fig. 7c). The synergistic nature of the afatinib/tozasertib combination was confirmed by multiple reference models^47^, including Bliss excess, Loewe, highest single-agent (HSA) and zero interaction potency (ZIP) (Fig. 3a; Supplementary Figs. 8, 9). Colony formation was nearly abolished following afatinib/tozasertib co-treatment (human cells: 37 nM each compound; murine cells: 111 nM each compound) (Fig. 3b). These findings were corroborated by growth curve assays (Fig. 3c), and were consistent across different *KRAS* mutations (G12C, G12S and G12D). Given that Aurora kinase inhibitors can display substantial cross-reactivity among family members, we decided to continue with the pan-AURK inhibitor.

### Dual targeting of ERBB and AURK promotes apoptotic cell death in *KRAS* mutant LUAD

**Fig. 4:**
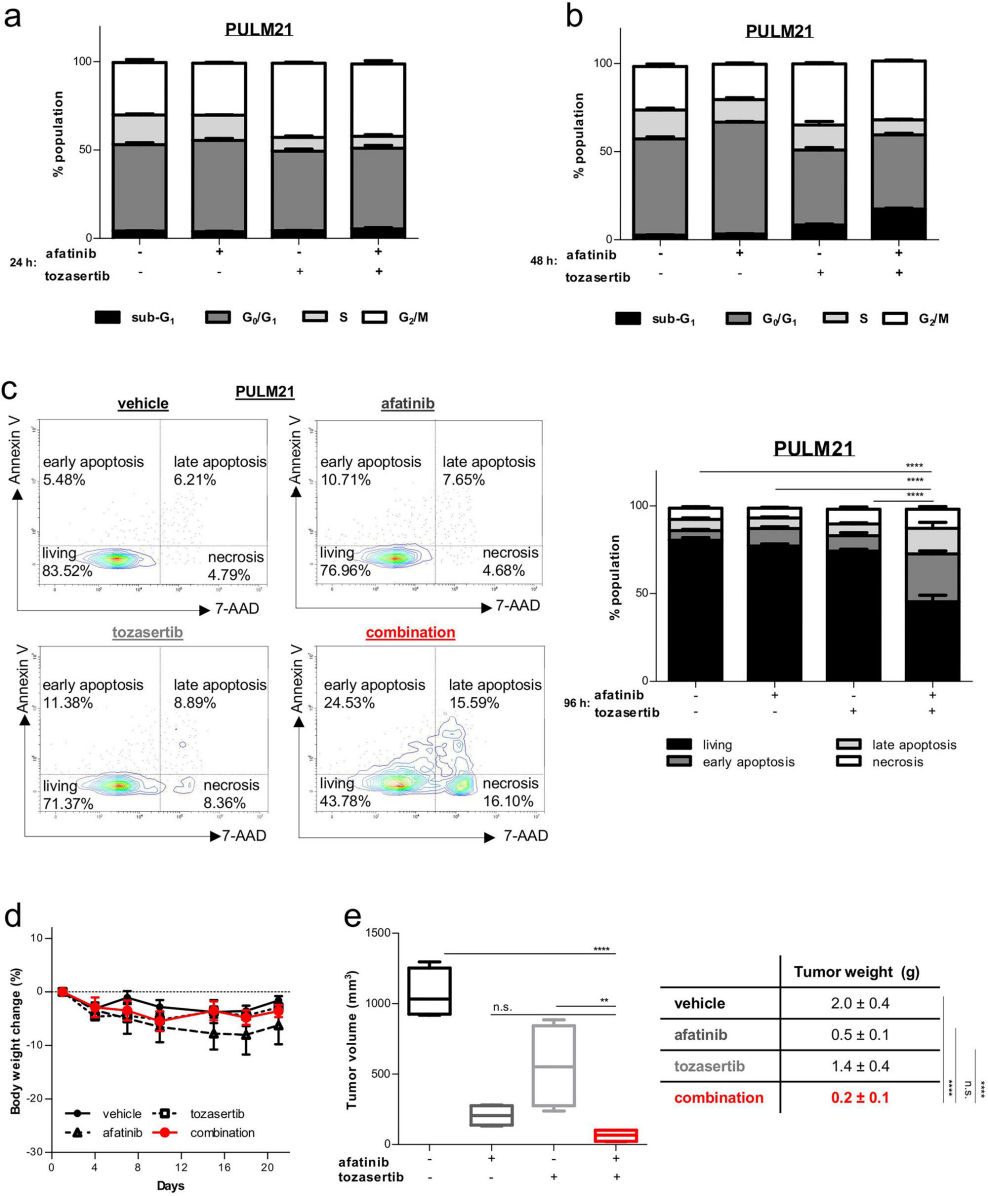
Combination of afatinib and tozasertib suppresses *KRAS* driven tumor development. **a** PULM21 cells were incubated with vehicle or the indicated compounds (37 nM each) for 24 hours, stained with propidium iodide, and analyzed by flow cytometry. Combination increases the proportion of cells in the G_2_ → M phase and induces apoptotic sub-G_1_ fraction. Bar graphs show cell cycle distribution. Analysis was performed in triplicate. **b** PULM21 cells were incubated with vehicle or the indicated compounds (37 nM each) for 48 hours, stained with propidium iodide, and analyzed by flow cytometry. Combination increases the proportion of cells in the G_2_ → M phase and induces apoptotic sub-G_1_ fraction. Bar graphs show cell cycle distribution. Analysis was performed in triplicate. **c** Treatment (37 nM each compound) – induced apoptosis was evaluated on day 4 by labeling PULM21 cells with annexin V/7-aminoactinomycin D (7-AAD) via fluorescence-activated cell sorting analysis. The percentage of cells in the upper left quadrant denotes cells that stained positive for annexin V only (early apoptosis). The cells in the upper right quadrant stained positive for annexin V and 7-AAD (late apoptosis). The percentage of cells in the lower right quadrant represents cells that stained positive for 7-AAD only (necrosis). Analysis was carried out in triplicate. Error bars indicate ± SEM. ****P < 0.0001 (One-way ANOVA with subsequent Bonferroni posttest). **d** PULM21 cells were subcutaneously injected into one flank of immunocompromised NSG recipients. Mice were treated once per day with vehicle, afatinib (20 mg/kg; p.o.), tozasertib (75 mg/kg; i.p.), or the combination (*n* = 4 mice for each group) on day 2 until terminal workup at day 21. Changes in body weight are presented. **e** Tumor volume and tumor weight at day 21 are presented. ** *P* < 0.01, **** *P* < 0.0001, n.s. not significant (One-way ANOVA with subsequent Bonferroni posttest).

Afatinib and tozasertib are known to block cell cycle at different phases. While the short-term treatment (24 h) induced some alterations (Fig. 4a), prolonged drug administration (48 h; (37 nM each compound for human cells; 111 nM each compound for murine cells) amplified the changes in cell cycle profiles. Inhibition of ERBB receptors alone, at a very low dose, gave rise to a pronounced increase in the number of cells in the G_0_ → G_1_ phase in PULM21 cells but not in MML416 cells (Fig. 4b; Supplementary Fig. 10b), which is consistent with the growth curve assay as these cells failed to respond to afatinib alone (Fig. 3c). On the other hand, AURK inhibition alone at a very low dose resulted in mitotic arrest in both cell lines (Fig. 4b and Supplementary Fig. 10b). Combination treatments enhanced the magnitude of response. Cells were not only accumulated in the G_2_ → M phase but also in the sub-G_1_ compartment, which represents dead cells (Fig. 4b; Supplementary Fig. 10a–c). The drug-induced toxicity of very low dose co-treatment stems from induction of apoptosis, as revealed by a significant increase in annexin V staining when compared to each monotherapy (37 nM for human cells; 111 nM for murine cells) (Fig. 4c; Supplementary Fig. 11a–d). We next evaluated therapeutic activity in vivo using a subcutaneous xenograft model. Immunocompromised mice transplanted with *KRAS* mutated tumor cells were treated daily with afatinib (20 mg/kg), tozasertib (75 mg/kg), or the combination starting two days after engraftment and continuing for 19 days. No changes in body weight were observed across treatment groups (Fig. 4d). In contrast to in vitro results, afatinib showed greater anti-tumor activity in vivo when compared with tozasertib (Fig. 4e). This difference likely reflects the longer treatment duration in vivo and the use of once-daily tozasertib dosing, instead of the recommended twice-daily schedule, to accommodate combination therapy in vivo and minimize potential adverse effects. The afatinib/tozasertib combination reduced tumor growth significantly compared to tozasertib and vehicle (Fig. 4e). While not statistically superior to afatinib alone, the combination consistently resulted in smaller tumors (Fig. 4e), suggesting a lethal interaction.

### Molecular mechanisms underlying ERBB/AURK inhibition

**Fig. 5:**
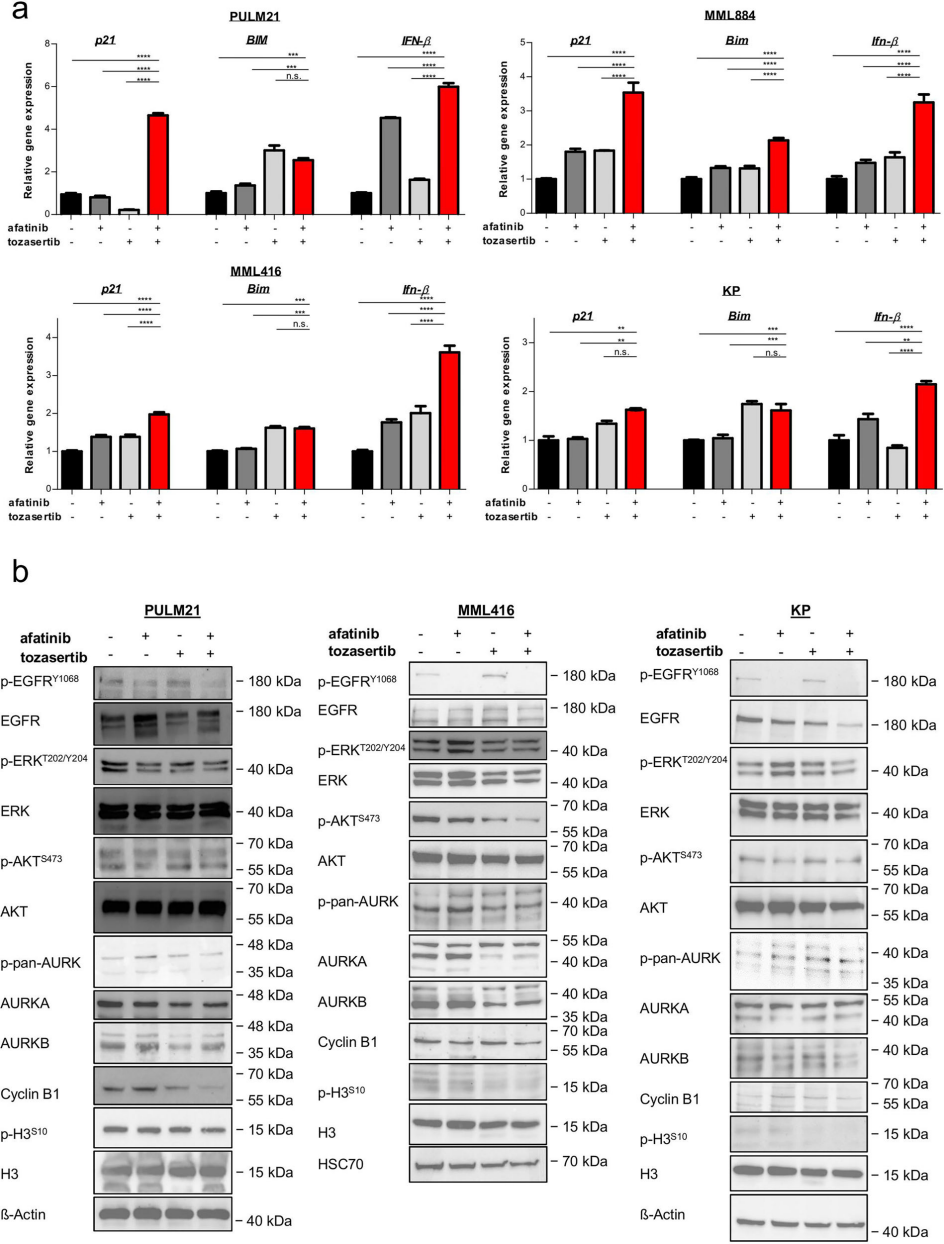
Signaling analysis upon afatinib/tozasertib co-treatment in *KRAS* mutated LUAD cells. **a** qRT-PCR for designated genes in cell lines treated with the indicated agents (37 nM each compound (PULM21); 111 nM each compound (MML884, MML416, KP)) for 96 h. Data are normalized against *28S* and presented as mean values ± SEM (*n* = 3). **P < 0.01, ***P < 0.001, ****P < 0.0001, n.s. not significant (One-way ANOVA with subsequent Bonferroni posttest). **b** Cells treated with the indicated agents (37 nM each compound (PULM21); 111 nM each compound (MML416, KP)) for 96 hours were assessed by immunoblots. ß-Actin and HSC70 served as a loading control.

To delineate downstream signaling mechanisms, we analyzed the expression and activation of key apoptotic and proliferative regulators. BIM, a pro-apoptotic effector implicated in TKI-induced cell death in *EGFR* mutant LUAD^43^ and tightly controlled by ERK signaling^43^, was induced by afatinib/tozasertib co-treatment at very low doses (37 nM in human cells; 111 nM in murine cells) in *KRAS* mutant LUAD cells (Fig. 5a). This increase coincided with reduced phosphorylation of both EGFR and ERK (Fig. 5b), consistent with ERK pathway suppression. Co-inhibition also elevated the expression of type I interferon *IFN-β* (Fig. 5a), a cytokine known to trigger apoptosis via STAT3 activation in polymorphonuclear cells and through AKT suppression in neuroblastoma^48,49^. In agreement, dual treatment resulted in STAT3 activation (PULM21 and KP) and a modest decrease in AKT phosphorylation (PULM21, MML416, KP) (Fig. 5b; Supplementary Fig. 12).

AURKB blockade has been linked to cell cycle arrest through induction of the CDK inhibitor p21^WAF1/CIP^ ^50^. Consistently, afatinib/tozasertib co-treatment at very low doses upregulated *p21*^WAF1/CIP^ in *KRAS* mutant cells, although effects on other cell cycle proteins varied by cell line (Fig. 5a; Supplementary Fig. 12). In PULM21 cells, *p21*^WAF1/CIP^ induction was accompanied by a mild rise in p38 phosphorylation, decreased levels of Cyclin D1, CDK6, and CDK4, and complete loss of phospho-RB, findings consistent with reports that AURKB inhibition disrupts E2F signaling via RB dephosphorylation, either directly or through p38 MAPK-mediated p21^WAF1/CIP^ induction^50,51^ (Supplementary Fig. 12a). By contrast, these effects could not be recapitulated in MML416 or KP cells (Supplementary Fig. 12b, c).

Moreover, combination therapy at a very low dose also reduced the levels of phospho-Histone H3 and Cyclin B1 (Fig. 5b), both markers of mitotic progression, suggesting mitotic defects and G_2_ → M arrest due to AURK inhibition^52^. Together, these data demonstrate that afatinib and tozasertib cooperate to engage both apoptotic and cell cycle regulatory programs, resulting in enhanced anti-tumor activity in *KRAS* mutant LUAD.

Notably, similar to EGFR-selective TKI^40,43,53^, afatinib triggered adaptive phosphorylation of AURK in three cell lines and TBK1 in two (consistent with its role in *KRAS* mutant cell survival^54^). Notably, the low-dose afatinib/tozasertib combination (37 nM in human; 111 nM in murine cells) suppressed these phosphorylation events in all tested lines (Fig. 5b; Supplementary Fig. 12). This suggests that co-inhibition disrupts feedback mechanisms promoting resistance.

**Fig. 6:**
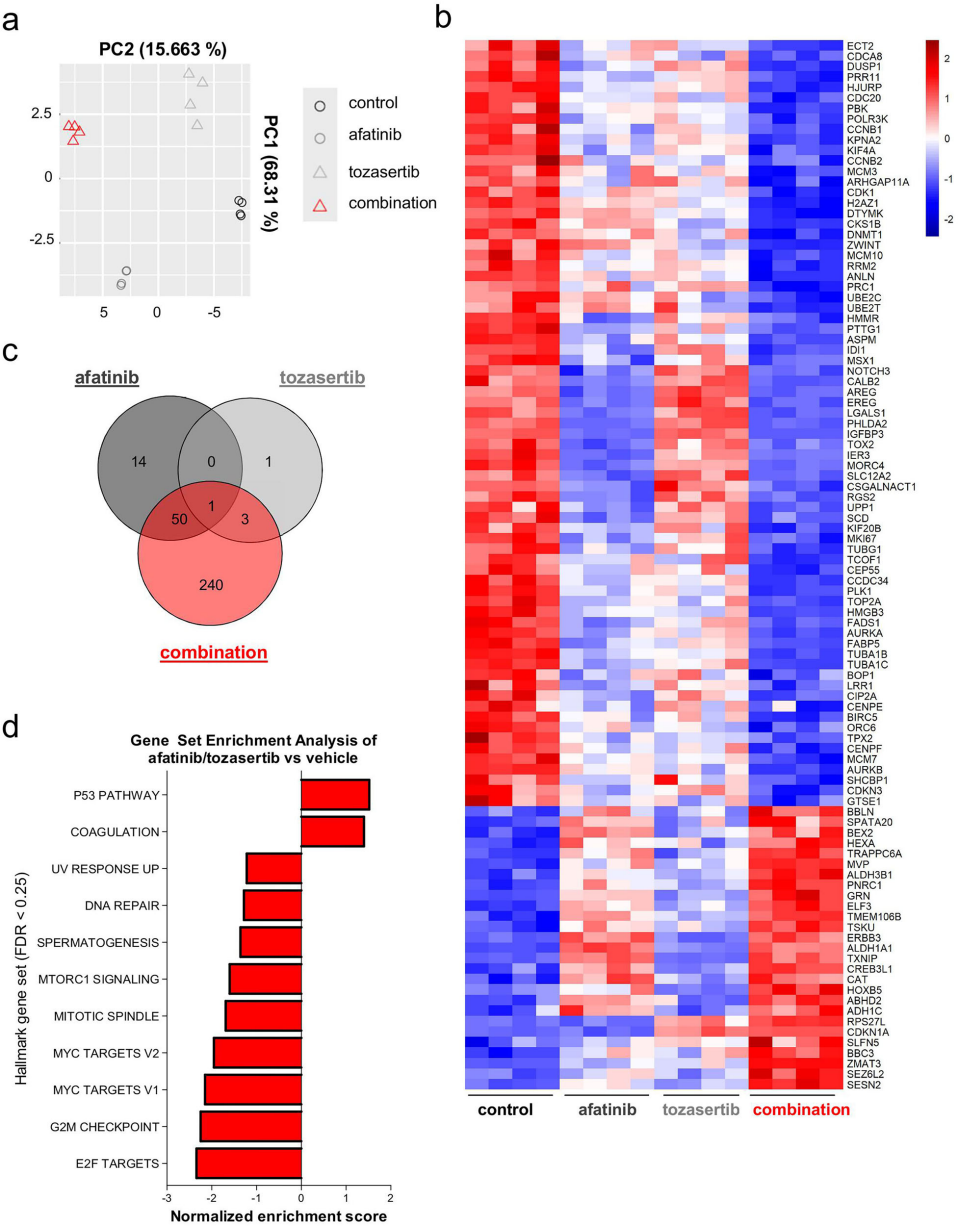
Transcriptomic profiling of *KRAS* mutated cells upon afatinib/tozasertib co-treatment. **a** Principal component analysis of RNA sequencing in PULM21 cells treated with the indicated agents (37 nM each compound) for 48 h is depicted. *n* = 4 per group. **b** Heat map shows the top 100 differentially expressed protein-coding genes (adjusted *P* ≤ 0.05; absolute log2-fold changes >1; contrast: afatinib/tozasertib vs vehicle). Regularized log-transformation of count data was used as input for the heat map. **c** A Venn diagram of differentially expressed genes upon combination treatment vs single agents (adjusted *P* ≤ .05; absolute log2-fold changes >1). **d** Overview of gene set enrichment analysis for the indicated contrast. The Hallmark Gene Set Collection was used as a reference. False discovery rate <0.25 was considered significant (NOM *P* < 0.05).

We next performed bulk RNA sequencing to examine mRNA expression profiles following 48 h co-treatment with afatinib and tozasertib (37 nM each). Principal component analysis revealed two dominant sources of variance: the effect of afatinib along PC1, accounting for 68.3% of total variance, and the effect of tozasertib along PC2, explaining 15.7% (Fig. 6a). Co-treatment with afatinib and tozasertib produced a distinct transcriptomic profile with altered expression of more than 200 protein-coding genes (Fig. 6b, c). GSEA revealed enrichment of apoptosis-related pathways and suppression of cell cycle programs, effects more pronounced with the combination than with monotherapies (Fig. 6d; Supplementary Fig. 13). Collectively, these findings mirror the observed cellular phenotype and nominate potential candidate biomarkers of drug response in future studies.

### Dual ERBB/AURK inhibition overcomes resistance to afatinib and sotorasib

To evaluate the therapeutic potential of combined ERBB and AURK inhibition beyond treatment-naïve contexts, we investigated its efficacy in an afatinib-resistant model. Afatinib-resistant PULM21 cells (PULM21-AR) were generated through stepwise dose escalation (Supplementary Fig. 14a). No secondary mutations were detected in the *EGFR* gene (data not shown). Compared to parental cells, PULM21-AR cells exhibited persistent activation of EGFR, ERK, and AURK signaling pathways (Supplementary Fig. 14b). Importantly, these resistant cells remained sensitive to tozasertib (Supplementary Fig. 14c), which suppressed these pathways (Supplementary Fig. 14d), highlighting a retained dependency that could be exploited therapeutically.

Interestingly, while AKT phosphorylation was markedly suppressed in PULM21-AR cells relative to the parental line, treatment with tozasertib led to a paradoxical increase in phospho-AKT levels. This observation suggests a compensatory feedback mechanism and underscores the complexity of signaling dynamics in resistant states. Nonetheless, these findings reinforce AURK inhibition as a promising strategy to overcome afatinib resistance in *KRAS* driven cancers.

**Fig. 7:**
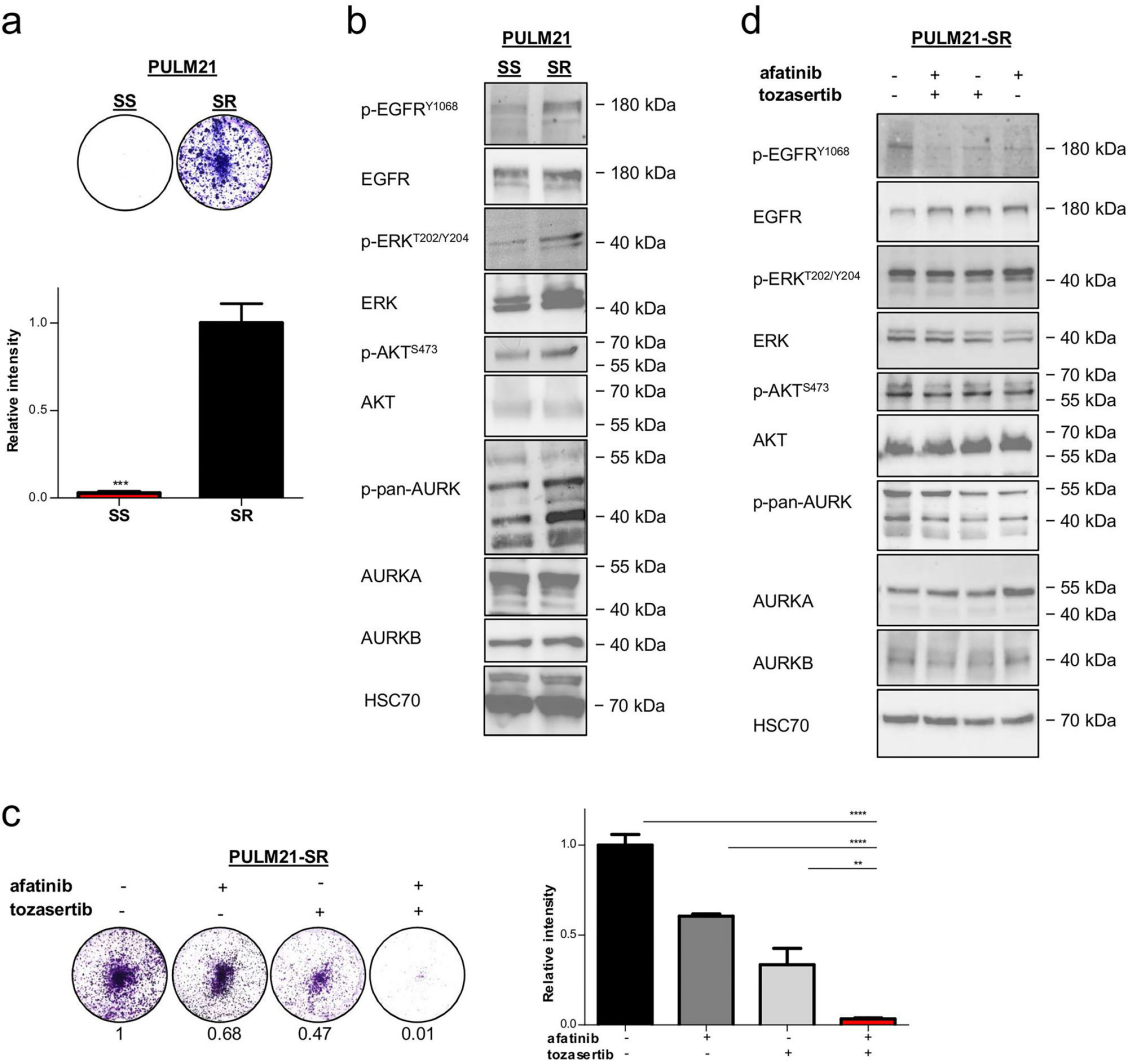
Sotorasib-resistant *KRAS* mutated cells remain sensitive to pan-ERBB/AURK inhibition. **a** Exposure to increasing concentrations of sotorasib over time conferred PULM21 cells resistant to the treatment (10 µM). Representative images of crystal violet staining of colonies after ten days are shown (*n* = 3 per group). ImageJ was used for quantification. Data are presented as mean values ± SEM. *** *P* < 0.001 (Student’s *t* test). Sensitive cells are indicated as SS; resistant cells are labeled with SR. **b** Changes in signaling upon resistance to sotorasib were assessed by immunoblot. HSC70 was used as a loading control. **c** PULM21-SR cells were treated with vehicle or the indicated drugs (37 nM each compound). Representative images of crystal violet staining of colonies after ten days are shown (*n* = 3 per group). ImageJ was used for quantification. Error bars indicate ± SEM. **P < 0.01, ****P < 0.0001 (One-way ANOVA with subsequent Bonferroni posttest). **d** PULM21-SR cells were treated with vehicle or the indicated agents (37 nM each compound) and assessed by immunoblot at 48 hours. HSC70 served as a loading control.

In a real-world setting, *KRAS* mutant LUAD patients who have undergone extensive prior treatment receive sotorasib as a second- or further-line therapy. However, the benefits are typically short-lived, highlighting the need for more durable therapeutic approaches. To model late-line therapy, sotorasib-resistant cells were generated by stepwise dose escalation (Fig. 7a), with no additional *KRAS* mutations detected (data not shown). Consistent with prior reports, these resistant cells displayed increased activation of EGFR, ERK, AKT and AURK signaling pathways (Fig. 7b), reflecting activation of bypass signaling routes^17,19,55^. Notably, afatinib/tozasertib co-treatment led to complete eradication of resistant clones (Fig. 7c) and suppression of EGFR and AURK phosphorylation (Fig. 7d). While AKT phosphorylation was modestly reduced with monotherapy, the combination treatment did not further enhance this effect (Fig. 7d). ERK phosphorylation remained unchanged across all treatment conditions. These findings indicate that the therapeutic effect in sotorasib-resistant cells is primarily mediated through EGFR and AURK inhibition and support the rationale for dual ERBB/AURK inhibition as a strategy to overcome sotorasib-resistance in *KRAS* mutant LUAD.

Collectively, our findings demonstrate that dual targeting of ERBB and Aurora kinases induces robust apoptosis, impairs adaptive signaling, and overcomes resistance in *KRAS* mutant LUAD, including models refractory to afatinib and sotorasib. These data establish a strong mechanistic rationale for clinical evaluation of ERBB/AURK combined inhibition in *KRAS* driven lung cancer.

## Discussion

*KRAS* mutations, long considered undruggable, have recently become actionable with the advent of selective inhibitors such as sotorasib (targeting KRAS-G12C) and investigational KRAS-G12D and pan-KRAS inhibitors^56–59^. However, despite initial clinical enthusiasm, responses have been limited and transient in *KRAS* driven LUAD. This is largely due to the rapid emergence of resistance driven by both genetic and non-genetic mechanisms. Such include reactivation of upstream receptor tyrosine kinases (e.g., EGFR), alternative signaling cascades (e.g., PI3K/mTOR, CDK4/6, AURK), and feedback-driven protein re-synthesis. This has prompted renewed interest in combinatorial therapeutic strategies that address tumor plasticity and adaptive resistance.

Our study builds on the growing recognition that ERBB family signaling is a key contributor in *KRAS* driven LUAD. We and others have shown that nascent *KRAS* mutant tumors depend on autocrine ERBB ligand production and that EGFR signaling remains active despite the presence of *KRAS* mutations^28,29^. While EGFR-selective inhibitors have historically failed in this setting due to compensatory activation of other ERBB members, pan-ERBB inhibitors such as afatinib and neratinib offer a broader blockade of this network. Encouragingly, the first clinical evidence for an afatinib-based regimen emerged in 2024: a patient with pneumonic-type *KRAS* mutated LUAD, refractory to multiple lines of therapy, achieved 14 months of stable disease without apparent adverse effects^60^. Nevertheless, monotherapies are rarely, if ever, curative. We therefore hypothesized that combining afatinib with agents that enhance apoptosis would improve therapeutic outcomes. Through unbiased high-throughput screening, we identified AURK inhibitors as potent synergistic partners with afatinib at low nanomolar doses. AURKs regulate key mitotic processes; their aberrant expression has been implicated in cancer progression, treatment resistance and genomic instability. Importantly, AURK inhibitors have non-overlapping side effects with ERBB inhibitors^61–63^, making them attractive for combination strategies. Co-treatment with afatinib and the pan-AURK inhibitor tozasertib significantly suppresses colony formation and growth in *KRAS* mutant models. Consistent with prior work showing synergy between EGFR-selective and AURKA-selective inhibitors in *KRAS* mutant LUAD^64^, our study confirms and extends these findings. We show that the synergistically lethal interaction is reproducible using pan-ERBB and pan-AURK inhibitors, thereby broadening the therapeutic options and enhancing clinical applicability. Importantly, we demonstrate that the afatinib/tozasertib combination retains potent activity in cells resistant to either afatinib or the KRAS-G12C inhibitor sotorasib, revealing a critical vulnerability in treatment-refractory disease. These results not only expand the therapeutic armamentarium for *KRAS* mutant LUAD but also provide a strong rationale for clinical evaluation in both KRAS-targeted therapy-naïve and resistant settings.

Mechanistically, afatinib treatment enhances AURK signaling, as shown by increased AURK phosphorylation, which is abrogated by tozasertib co-treatment. This points to AURK signaling as a bypass survival mechanism under ERBB blockade. Dual ERBB/AURK inhibition results in robust apoptosis induction, mediated in part through BIM. Recent studies have established that BIM, a pro-apoptotic BH3-only protein, integrates signals ERK and AURK inputs; dual suppression reduces BIM phosphorylation, stabilizing the protein and triggering cell death^40,42,43^. Co-treatment at very low doses also arrests the cell cycle via *p21*^WAF1/CIP^ induction, acting independently of p53 and likely influenced by p38 or other regulators (e.g., FoxO, BRCA1, MITF, NPM1, p16), warranting further study. Although effects on other cell cycle proteins differ across lines (as expected consequence of cellular and tumor heterogeneity), the combination consistently drives cell death.

Co-treatment with the ERBB inhibitor afatinib and the AURK inhibitor tozasertib enhanced STAT3 phosphorylation and IFN-β expression. Although STAT3 activation is often linked to survival, sustained activation, particularly alongside IFN-β induction, can promote apoptosis^48^. These effects may reinforce tumor cell-intrinsic death programs and enhance tumor immunogenicity, consistent with reports of AURK inhibition-driven innate immune activation via type I IFN signaling^69^, a hypothesis warranting future investigation. The combination also reduced phospho-TBK1 to baseline or below in all tested cell lines, suppressing additional early bypass signaling.

Collectively, the combination therapy targets several complementary pathways to promote tumor cell death. Afatinib/tozasertib blocks MAPK and AKT signaling, and inhibits AURK, while increasing p21^WAF1/CIP^, BIM and IFN-β. These changes enhance apoptotic priming and may foster a more immunogenic tumor environment. Together, they converge to suppress tumor growth, amplify apoptosis, and limit adaptive resistance, supporting the mechanistic basis for the synergistic efficacy of the afatinib/tozasertib combination.

While clinical trials are currently exploring combinations such as afatinib and sotorasib (NCT04185883), our data suggest that the afatinib/tozasertib combination may offer an even more effective strategy, particularly for overcoming acquired resistance to KRAS inhibition. Importantly, cells resistant to either afatinib or sotorasib as monotherapy remain sensitive to the combination afatinib/tozasertib, underscoring the concept that targeting parallel and compensatory signaling pathways, namely ERBB family receptors and Aurora kinases, can circumvent mechanisms of adaptive resistance. Moreover, the distinct mechanisms of action and non-overlapping toxicity profiles of afatinib and tozasertib enhance the clinical feasibility of this approach. The ability of the afatinib/tozasertib combination to eradicate sotorasib-resistant clones highlights a critical vulnerability in *KRAS* driven tumors that persist despite direct KRAS inhibition. This is particularly significant given the modest clinical performance of sotorasib, which has failed to demonstrate a meaningful overall survival benefit and has achieved only a marginal improvement in progression-free survival in heavily pretreated patients (PFS 5.6 months (sotorasib) vs 4.5 months (docetaxel)). These disappointing outcomes reflect the rapid emergence of resistance mechanisms, often driven by bypass pathway activation. By simultaneously disabling ERBB-mediated signaling and mitotic control via AURK inhibition, the afatinib/tozasertib combination effectively suppresses key survival pathways, offering a compelling rationale for clinical evaluation as a strategy to enhance the durability of responses and improve outcomes in *KRAS* mutant lung adenocarcinoma.

Despite these promising findings, several limitations should be acknowledged. First, the in vivo experiments were conducted in a very small cohort (n = 4 per group), limiting statistical power. However, tumor volumes in the combination group were consistently smaller than with either monotherapy, suggesting a biological effect worth further evaluation. Tozasertib was administered once daily instead of the recommended twice-daily schedule, likely underestimating its activity in vivo. Pharmacokinetic and pharmacodynamic studies will be needed to define optimal dosing, exposure and sequential application. Second, both afatinib and tozasertib have nontrivial toxicities - afatinib mainly gastrointestinal and dermatologic, and tozasertib associated with myelosuppression, gastrointestinal intolerance, and fatigue. While clinically relevant, they are generally manageable with dose optimization, intermittent or lower-dose regimens, close monitoring, proactive supportive care, and early intervention strategies. Importantly, in our xenograft model the combination did not worsen toxicity compared to either agent alone, suggesting tolerability. Finally, as this preclinical work relied on cell line models, which cannot fully capture the complexity of human tumors, validation in genetically engineered and patient-derived systems will be essential. For clinical translation, carefully designed trials, incorporating flexible dosing schedules, robust pharmacodynamic endpoints, close safety monitoring, and biomarker-guided patient selection to balance efficacy with tolerability, will be essential to confirm this regimen as a rational salvage option for treatment-refractory *KRAS* mutant LUAD.

In summary, this study provides compelling preclinical evidence that dual inhibition of ERBB and AURK pathways represents a promising therapeutic strategy for *KRAS* mutant LUAD. The ability of this combination to override resistance, activate apoptosis, and potentially enhance immune system engagement underscores its clinical potential. Future clinical trials should evaluate this strategy not only in lung cancer but also in other *KRAS* mutant malignancies, including pancreatic and colorectal cancers, particularly in settings where conventional KRAS-targeted strategies fail. Furthermore, the modulation of candidate genes of interest such as BIM may guide treatment response monitoring and inform patient stratification. Overall, our study provides a mechanistic framework for exploiting synthetic vulnerabilities in oncogene-driven cancers and reinforces the value of systems-based therapeutic approaches in precision oncology.

## Methods

### Cell culture

*KRAS* mutated NSCLC adenocarcinoma cell lines (human: PULM21, PULM24, A549, A427 and murine: 368T1, MML416, MML884, KP) were cultured under standard conditions in RPMI-1640 (Gibco) supplemented with 10% fetal bovine serum and 1% penicillin/streptomycin (Gibco). Cells were maintained at 37 °C in a humidified incubator containing 5% CO_2_. MML416, MML884, PULM21 and PULM24 were a gift from Monica Musteanu (CNIO, Spain). 368T1 cells were a gift of Dr. Tyler Jacks^65^. PULM21 and PULM24 cells were derived from the PDX model and harbor a *KRAS*-G12C mutation. The KP cell line was isolated in-house from respective mice by enzymatic digestion of lungs^28^. Cell numbers were counted automatically with the DeNovix CellDrop™; viability was measured by CellTiterGlo (Promega) according to the manufacturer’s instructions.

To establish drug resistance, PULM21 cells were cultured in increasing concentrations of each compound over two months. Development of resistance was determined in a colony formation assay using parental and resistant cells in the absence and presence of appropriate drugs.

### Drug screening

High-throughput screening was performed using an automated 384-well platform. 368T1 and PULM24 were screened against a total of 2097 compounds from seven different compound collections in the absence and presence of afatinib (1 µM): (i) NIH clinical collection (10 µM), CeMM library of unique drugs (10 µM), collection of anti-cancer drugs (20 µM), natural products (10 µM), epigenetic compounds (ranging from 10 to 50 µM), Sigma CPR library (10 µM) and kinase inhibitor library (10 µM). Adenosine triphosphate (ATP) levels were measured after 72 hours as a surrogate for cell viability (CellTiterGlo). Data for each cell line were normalized to the 32 negative control (DMSO) wells on each plate (100% viability) and the 32 positive control wells (20 µM bortezomib; set to 0% viability). A Z’-factor^66^ was calculated for each 384-well plate individually.

For synergy matrices, cells were plated in triplicate in 96-well plates. ATP content was measured after 72 or 96 hours by using CellTiterGlo according to the manufacturer’s instructions. The percentage deviation from the Bliss independency model^32^ was determined via the following formula: E_xy_ = E_x_ + E_y_ − (E_x_E_y_). E represents the effect on viability of drugs x and y expressed as a percentage of the maximum effect. Other reference models were tested using SynergyFinder 3.0 online tool^47^. Afatinib and BI-31266 were manufactured by Boehringer Ingelheim. Tozasertib, alisertib and sotorasib were purchased from Selleckchem and MedChem Express, respectively.

### Immunoblot analysis

Cells were harvested using a lysis buffer containing 20 mM Tris, 100 mM NaCl, 1 mM Na_3_VO_4_, 100 mM NaF, 20 mM glycerol 2-phosphate, 2.5 mM EDTA, 1 mM EGTA, 1% Nonidet P-40, and 1 mM PMSF, and Complete Protease Inhibitor Tablets (Roche). Lysates were loaded on 8–12% polyacrylamide gels (as appropriate), separated using an SDS-containing running buffer, and transferred to nitrocellulose membranes (Invitrogen) by semi-dry blotting. After blocking (5% BSA or milk in 1x TBS-T for 1 h at room temperature), membranes were probed with primary antibodies against phospho-EGFR (Y1068, Cell Signaling #2234), EGFR (Cell Signaling #4267), phospho-ERK1/2 (T202/Y204, Cell Signaling #4376), ERK1/2 (Cell Signaling #4695), phospho-Histone H3 (Ser10, Cell Signaling #9701), Histone H3 (Cell Signaling #4499), Cyclin B1 (Santa Cruz, sc-7393), phospho-Aurora A /Aurora B/ Aurora C (T288 /T232 /T198, Cell Signaling #2914), Aurora A (Santa Cruz sc-56881), Aurora B (Cell Signaling #3094), phospho-STAT3 (Y705, Cell Signaling #9131), STAT3 (Cell Signaling #9139), phospho-TBK1 (S172, Cell Signaling #5483), TBK1 (Cell Signaling #3504), phospho-p38 (T180/Y182, Cell Signaling #4511), p38 (Cell Signaling #8690), CDK6 (Santa Cruz sc-56282), CDK4 (Santa Cruz sc-23896), Cyclin D1 (Santa Cruz sc-8396), RB (Santa Cruz sc-102), phospho-RB (S807/811, Cell Signaling 9308), phospho-AKT (S473, Cell Signaling #9271) and AKT (Cell Signaling #9272). HSC70 (Santa Cruz sc-7298) and ß-Actin (Santa Cruz sc-47778) were used as a loading control.

### Clonogenic assay

Cells were seeded on six-well plates in triplicate and cultured in the absence and presence of drug for 7–10 days until clear colonies were formed. Culture medium with fresh drugs was exchanged every 2 days. Colonies were fixed with 3.7% formaldehyde and stained with 0.1% crystal violet.

### Cellular growth measurement

For growth over time experiments, cells treated with inhibitor or vehicle control were counted and reseeded every 4 days. Proliferation rates were presented by cumulative cell numbers.

Cell cycle profiles upon drug treatment were obtained by staining cells with propidium iodide (50 μg/mL) in hypotonic lysis solution (0.1% (w/v) sodium citrate, 0.1% (v/v) Triton X-100, 100 μg/mL RNAse) and incubating at 37 °C for 30 min before measurement via FACS.

### Apoptosis measurement

Apoptosis induction upon drug treatment was evaluated by staining cells with AnnexinV FITC (eBioscience, ThermoFisher Scientific) and 7-amino-actinomycin D (7-AAD) Viability Staining Solution (Biolegend; excitation at 488 nm) according to the manufacturer’s instructions, followed by FACS analysis.

### Quantitative measurement of mRNA

RNA of cells was purified using E.Z.N.A. total RNA kit (Omega Biotek) and reverse transcribed using iScript cDNA synthesis kit (Bio-Rad). Quantitative real-time PCR was performed employing the GoTaq® qPCR Master Mix (Promega). Analysis was carried out in triplicate, using *28S* as a control gene. The primer sequences will be supplied upon request.

### RNA sequencing and data analysis

Cells were treated with drugs (37 nM each compound) or vehicle control for 48 hours; RNA was isolated using Qiagen RNeasy Mini Kit followed by DNAse treatment. NGS libraries from total RNA samples were prepared at the Biomedical Sequencing Facility of the CeMM Research Center for Molecular Medicine of the Austrian Academy of Sciences using QuantSeq 3’ mRNA-Seq V2 Library Prep Kit with the UDI kit (Lexogen GmbH, Vienna, Austria). Resulting library concentrations were quantified with the Qubit® 1X dsDNA HS Assay Kit (Q32856, Thermo Fisher Scientific, Waltham, MA, USA) and the fragment size distribution was assessed using the High Sensitivity DNA Kit (5067-4626, Agilent, Santa Clara, CA, USA) on a 2100 Bioanalyzer High-Resolution Automated Electrophoresis instrument (G2939A/B, Agilent). Before sequencing, sample-specific NGS libraries were diluted and pooled in equimolar amounts. Expression profiling libraries were sequenced on a NovaSeq 6000 instrument (Illumina, San Diego, CA, USA) following a 100-base-pair, single-end recipe. Raw data acquisition (NovaSeq Control Software, NCS, 1.8.1) and base calling (Real-Time Analysis Software, RTA, 3.4.4) were performed on-instrument, while the subsequent raw data processing off the instruments involved two custom programs based on Picard tools (2.19.2). In a first step, base calls were converted into lane-specific, multiplexed, unaligned BAM files suitable for long-term archival (IlluminaBasecallsToMultiplexSam, 2.19.2-CeMM), thereby annotating an eventual start of NGS adapter sequences in XT tags. In a second step, archive BAM files were demultiplexed into sample-specific, unaligned BAM files (IlluminaSamDemux, 2.19.2-CeMM), annotating unique molecular index (UMI) sequences in RX tags and UMI base quality scores in QX tags. NGS reads were mapped to the Genome Reference Consortium GRCh38 assembly via “Spliced Transcripts Alignment to a Reference” (STAR, 2.7.11b)^67^ utilising the “basic” GENCODE transcript annotation from version v46 (May 2024) as reference transcriptome. Since the hg38 assembly flavour of the UCSC Genome Browser was preferred for downstream data processing with Bioconductor packages for entirely technical reasons, GENCODE transcript annotation had to be adjusted to UCSC Genome Browser sequence region names. STAR was run with options recommended by the ENCODE project. Metadata annotation, such as the start of NGS adapter sequences (in XT tags), or unique molecular index (UMI) sequences (in RX tags) and UMI base quality scores (in QX) tags were propagated from unaligned BAM files to the aligned BAM files via Picard MergeBamAlignment and duplicated alignments were marked with Picard MarkDuplicates in a UMI-aware manner but not removed. The differential expression modelling was based on R (4.4.3) and Bioconductor (3.20). NGS read alignments overlapping Ensembl exon features were counted with the Bioconductor GenomicAlignments (1.42.0) package via the summarizeOverlaps function in Union mode, ignoring secondary alignments, alignments not passing vendor quality filtering and for UMI-supporting protocols only, also duplicate alignments. Since the QuantSeq 3’ mRNA-seq FWD protocol leads to sequencing of the second strand, alignments were counted strand-specifically in feature (i.e., gene, transcript, and exon) orientation. Exon-level counts were aggregated to gene-level counts, and the Bioconductor DESeq2^68^ (1.46.0) package was used to test for differential expression based on a model using the negative binomial distribution.

### Sanger sequencing

For analysis of the *EGFR* and *KRAS* mutational status, the respective coding sequence was amplified using the following primer pairs:

*EGFR*_1

Fw: 5´-CCCTGACTCCGTCCAGTATTG-3´

Rv: 5´-GCTTCGTCTCGGAATTTGCG-3´

*EGFR*_2

Fw: 5´-AGTGACTGCTGCCACAACC-3´

Rv: 5´-GCACTTGTCCACGCATTCC-3´

*EGFR*_3

Fw: 5´-AGGTCTGCCATGCCTTGTG-3´

Rv: 5´-TGTTGGCTTTCGGAGATGTTGC-3´

*EGFR*_4

Fw: 5´-GCGTTCGGCACGGTGTAT-3´

Rv: 5´-TGTCTTCTTCATCCATCAGGGCA-3´

*EGFR*_5

Fw: 5´-GATGATAGACGCAGATAGTCGCC-3´

Rv: 5´-TGGGATGGAGGACCTGCT-3´

*KRAS*

Fw: 5´-TTTCGGACTGGGAGCGAG-3´

Rv: 5´-GCTAACAGTCTGCATGGAGCA-3´

PCR products were purified using the FavorPrep™ PCR Clean-Up Mini Kit (FAVORGEN BIOTECH CORP) and Sanger-sequenced by LGC Genomics.

### Mouse xenograft model

Mice were maintained under special pathogen-free conditions at the Institute of Pharmacology, Medical University of Vienna. Animal husbandry and experimental protocols as described below followed ethical guidelines and were approved by the Austrian Federal Ministry of Science, Research and Economy. 1 x 10^6^ PULM21 cells were resuspended in a 2:1 mixture of PBS/Matrigel (100 µl:50 µl) on ice. The mixture was subcutaneously inoculated into one flank of NOD/SCID/IL-2Rγ^−/−^ (NSG) mice. The animals were then randomized to receive afatinib (20 mg/kg; oral gavage), tozasertib (75 mg/kg; intraperitoneal), the combination or vehicle control. The mice were dosed 5 times per week starting on day 2 after engraftment, until terminal workup on day 21. Tumor volume was measured using a caliper and calculated using the formula [(length x width^2^)/2].

### Statistical analysis

Statistical analysis was carried out by using a two-tailed unpaired Student’s *t* test, a one-way ANOVA with subsequent Bonferroni posttest (comparing the combination with vehicle and monotherapies) or Mann-Whitney test as appropriate. Statistically significant differences are indicated by asterisks as follows: *P < 0.05, **P < 0.01, and ***P < 0.001, and ****P < 0.0001. Data are presented as mean values ± standard error of the mean (SEM) and were analyzed by using GraphPad software.

## Supplementary information

revSupplementary material_IUJ

## Data Availability

The RNA sequencing datasets generated and/or analyzed during the current study will be published on the ArrayExpress archive (accession number: E-MTAB-16181). Detailed drug screen results can be provided by the corresponding author upon justified request. All other data are available in the main text or the supplementary materials.
