# Supplementary material for: KRAS mutated lung adenocarcinoma responds to pan-ERBB and Aurora kinase inhibitors
Source: NPJ Precis Oncol. 2026 Jan 12;10:14. doi: 10.1038/s41698-025-01242-8 (PMC12796156; doi:10.1038/s41698-025-01242-8)

Supplementary material

KRAS mutated lung adenocarcinoma responds to pan-ERBB and Aurora kinase inhibitors

Iris Z. Uras[1*], Marija V. Trkulja[1+], Abdelrahman K.A.A. Salama[1+], Jaqueline Horvath[1], Khushi Asnani[1], Christoph Trenk[1], Stefan Kubicek[2], Martin Bilban[3], Herwig P. Moll[1], Emilio Casanova[1,4]

Contents

Figure S1. Overview of the compounds used in the high-throughput drug screening in *KRAS* mutated LUAD cells

Figure S2. Overview of growth inhibition by various pathway inhibitors ± afatinib

Figure S3. Overview of the agents used in pairwise drug combination viability assays

Figure S4. Pairwise drug combination viability screen reveals potential synergistic partners of afatinib

Figure S5. Bliss prediction model of afatinib in combination with inhibitors of Aurora kinase and IGF1R

Figure S6. Levels of *AURKA* and *AURKB* are elevated in lung adenocarcinoma

Figure S7. Co-inhibition of ERBB with AURKA or AURKB suppresses the viability of *KRAS* mutated LUAD cells

Figure S8. Synergy score analysis of combined ERBB and AURK inhibition in PULM21 and MML416 cells

Figure S9. Synergy score analysis of combined ERBB and AURK inhibition in MML884 and KP cells

Figure S10. Combined ERBB and AURK inhibition induces cell cycle arrest in *KRAS* mutant cells

Figure S11. AURK inhibition enhances afatinib-induced apoptosis in LUAD cells with *KRAS* mutations

Figure S12. Signaling analysis upon afatinib/tozasertib co-treatment in *KRAS* mutated LUAD cells

Figure S13. Comparative gene set enrichment analysis upon afatinib and tozasertib exposure in PULM21 cells

Figure S14. Afatinib-resistant *KRAS* mutated cells remain sensitive to AURK inhibition

Figure legends

Supplementary references

Western Blot original data

a

Collection	Concentration
NIH clinical collection	10 µM (0.2% DMSO)
CeMM library of unique drugs	10 µM (0.2% DMSO)
Anti-cancer drugs	20 µM (0.2% DMSO)
Natural products	10 µM (0.2% DMSO)
Epigenetic compounds	10 - 50 µM (0.2% DMSO)
Sigma CPR library	10 µM (0.2% DMSO)
Kinase inhibitor library	10 µM (0.2% DMSO)

b

Pathway	Target	# Compound
Signaling	PI3K/PDK1/AKT/mTOR	30
	RTK	37
	TK	8
	STK	13
	MAPK/MEK/ERK	18
	JAK/STAT	5
	NF-kB	4
	MET	1
Cell Cycle	CDK	10
	AURK	2
	PLK1	3
	Kinesin	2
DNA damage	CHK	2
	ATM	1
	DNA-PK	1
Cell adhesion/migration	DDR1	1
	FAK	3
	ROCK	2

Others	# Compound
Anti-cancer	27
Microbiology	26
Epigenetic modulators	17
Anti-inflammatory/-immunomodulatory	8
Apoptosis-inducers	3
Metabolism	16
Antioxidant	5
Antiemetic	2
Antiepileptic	5
Central nervous system	8
Anticoagulant	2
De novo synthesis	4
Antihistamine	3
Glutamate modulators	3
Vessels	8
Hypoxia	1
Proteasome inhibitor	1
Natural products, muscle relaxers, opioid receptors, chelating agents, mimetics etc	31

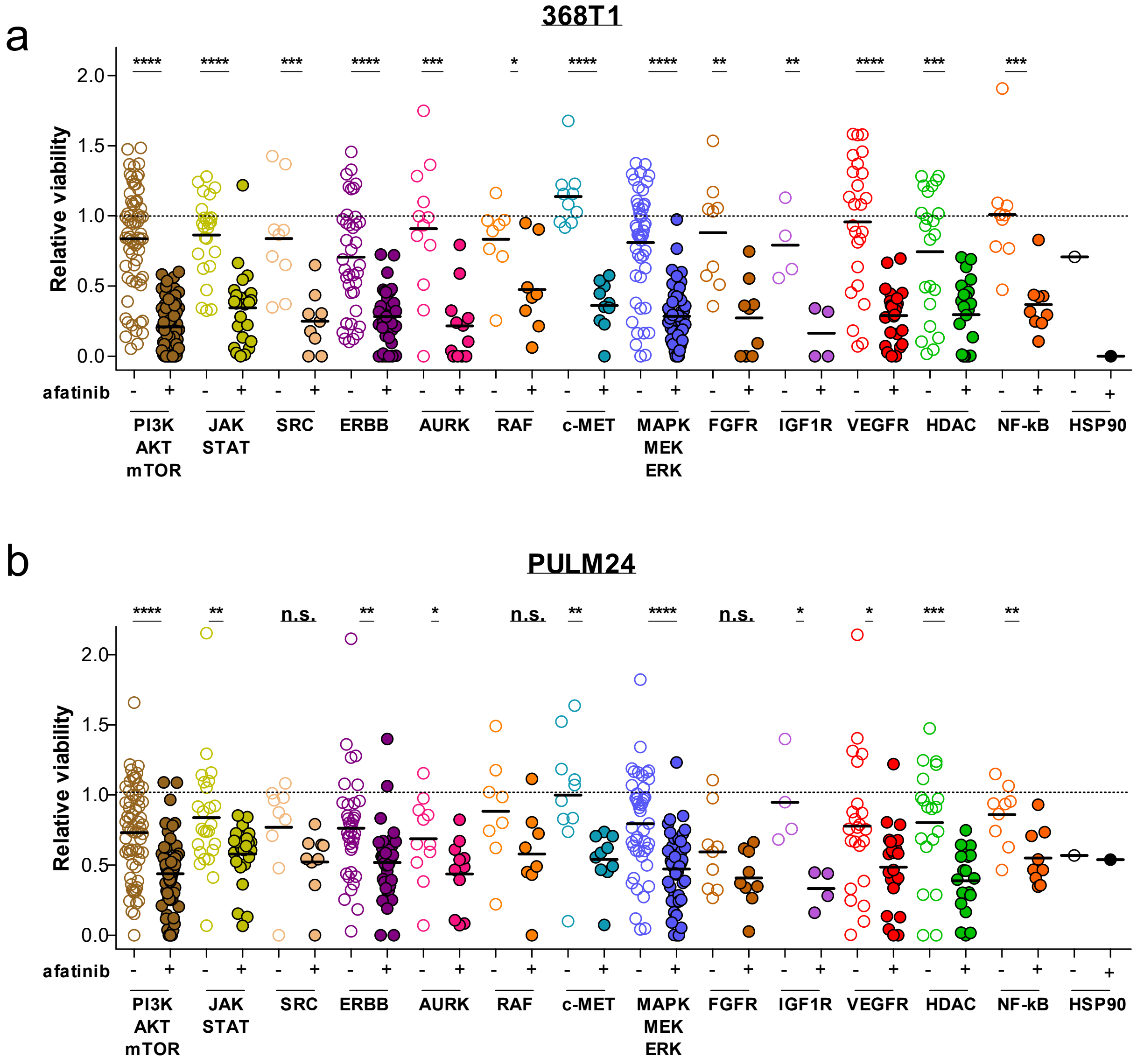
a
368T1
Relative viability
2.0
1.5
1.0
0.5
0.0
**** **** *** **** *** * **** **** ** ** **** *** ***
afatinib
- + - + - + - + - + - + - + - + - + - + - + - + - + - +
PI3K AKT mTOR
JAK STAT
SRC
ERBB
AURK
RAF
c-MET
MAPK MEK ERK
FGFR
IGF1R
VEGFR
HDAC
NF-kB
HSP90
b
PULM24
Relative viability
2.0
1.5
1.0
0.5
0.0
**** ** n.s. ** * n.s. ** **** n.s. * * *** **
afatinib
- + - + - + - + - + - + - + - + - + - + - + - + - + - +
PI3K AKT mTOR
JAK STAT
SRC
ERBB
AURK
RAF
c-MET
MAPK MEK ERK
FGFR
IGF1R
VEGFR
HDAC
NF-kB
HSP90

a

	368T1	A427	A549	PULM24
**Origin**	Murine	Human	Human	Human
**NSCLC histology**	Adenocarcinoma	Adenocarcinoma	Adenocarcinoma	Adenocarcinoma
KRAS	G12D	G12D	G12S	G12C
p53	null	wild-type	wild-type	null

b

Compound	Target	368T1	A427	A549	PULM24
**(S)-(+)-Camptothecin**	Topoisomerase I	+	-	-	-
**Apitolisib**	mTOR	+	-	-	-
**AZD 7762**	CHK	+	-	-	-
**BI 2536**	PLK1	+	+	-	-
**BMS-754807**	IGF1R	+	+	+	+
**Briciclib**	eIF4E	-	+	-	-
**Cabozantinib**	VEGFR	+	-	-	-
**CAY10626**	PI3K/mTOR	+	+	-	-
**Crizotinib**	ALK	+	-	-	-
**Danusertib**	AURK	+	+	+	+
**Foretinib**	c-MET/VEGFR	+	-	-	-
**GSK1838705A**	ALK	+	-	+	+
**LY2835219 mesylate**	CDK	-	-	+	-
**NVP-AEW541 (hydrochloride)**	IGF1R	+	-	+	-
**OSI-906**	IGF1R	+	-	+	-
**Pimasertib**	MEK	+	+	-	-
**SB715992**	Kinesin	+	-	-	-
**SB743921**	Kinesin	+	-	-	-
**TAK901**	AURK	+	-	-	-
**CAY10621**	SPHK1	-	-	-	-
**L-threo-Sphingosine C-18**	PKC	-	-	-	-
**N,N-Dimethylsphingosine**	SPHK	-	-	-	-
**Nintedanib**	VEGFR/FGFR/PDGFR	-	-	-	-
**SKI II**	SPHK	-	-	-	-
**Volasertib**	PLK1	-	-	-	-

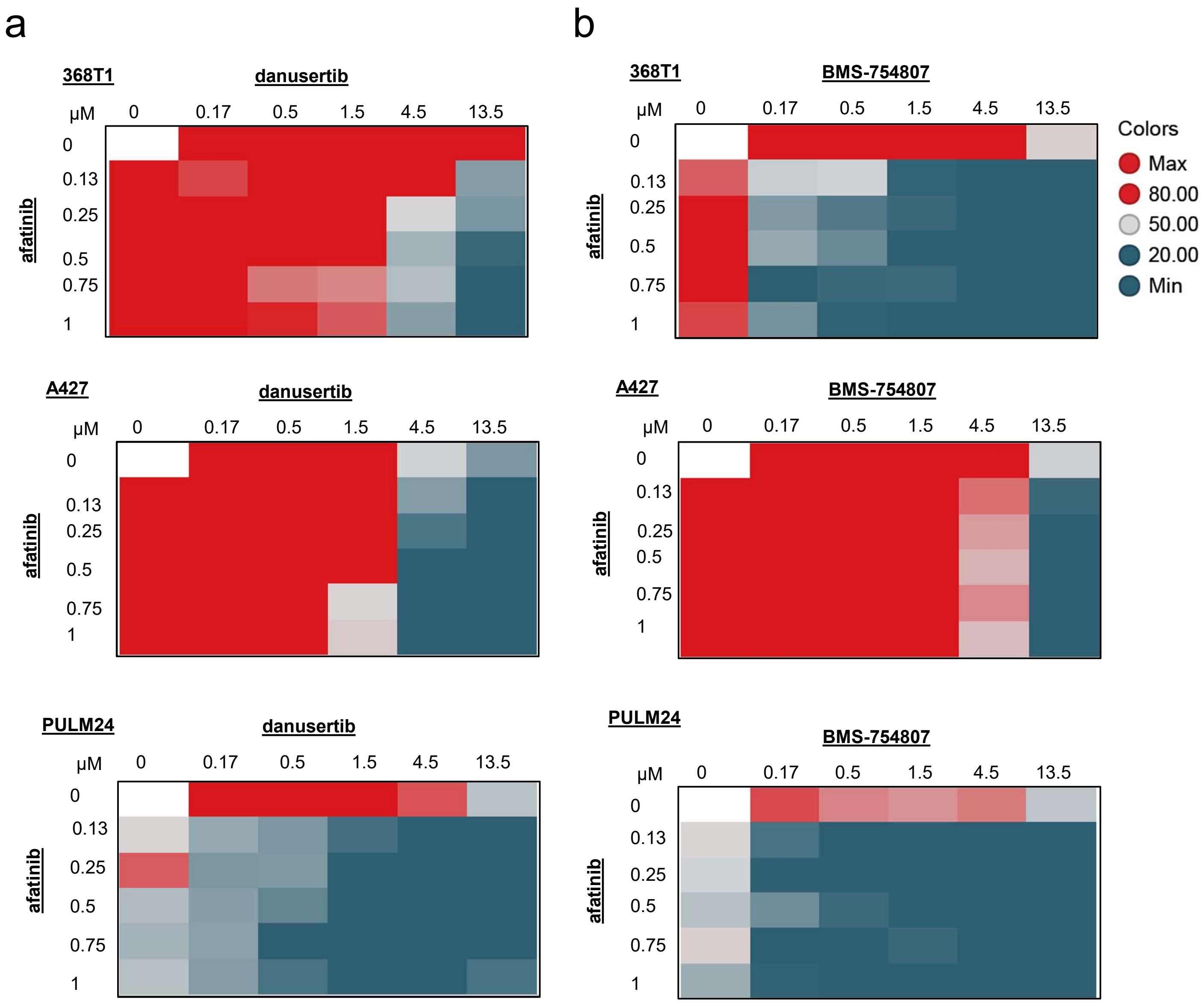

a
368T1
danusertib
µM
0
0.17
0.5
1.5
4.5
13.5
afatinib
0
0.13
0.25
0.5
0.75
1
A427
danusertib
PULM24
danusertib
b
368T1
BMS-754807
A427
BMS-754807
PULM24
BMS-754807
Colors
Max
80.00
50.00
20.00
Min

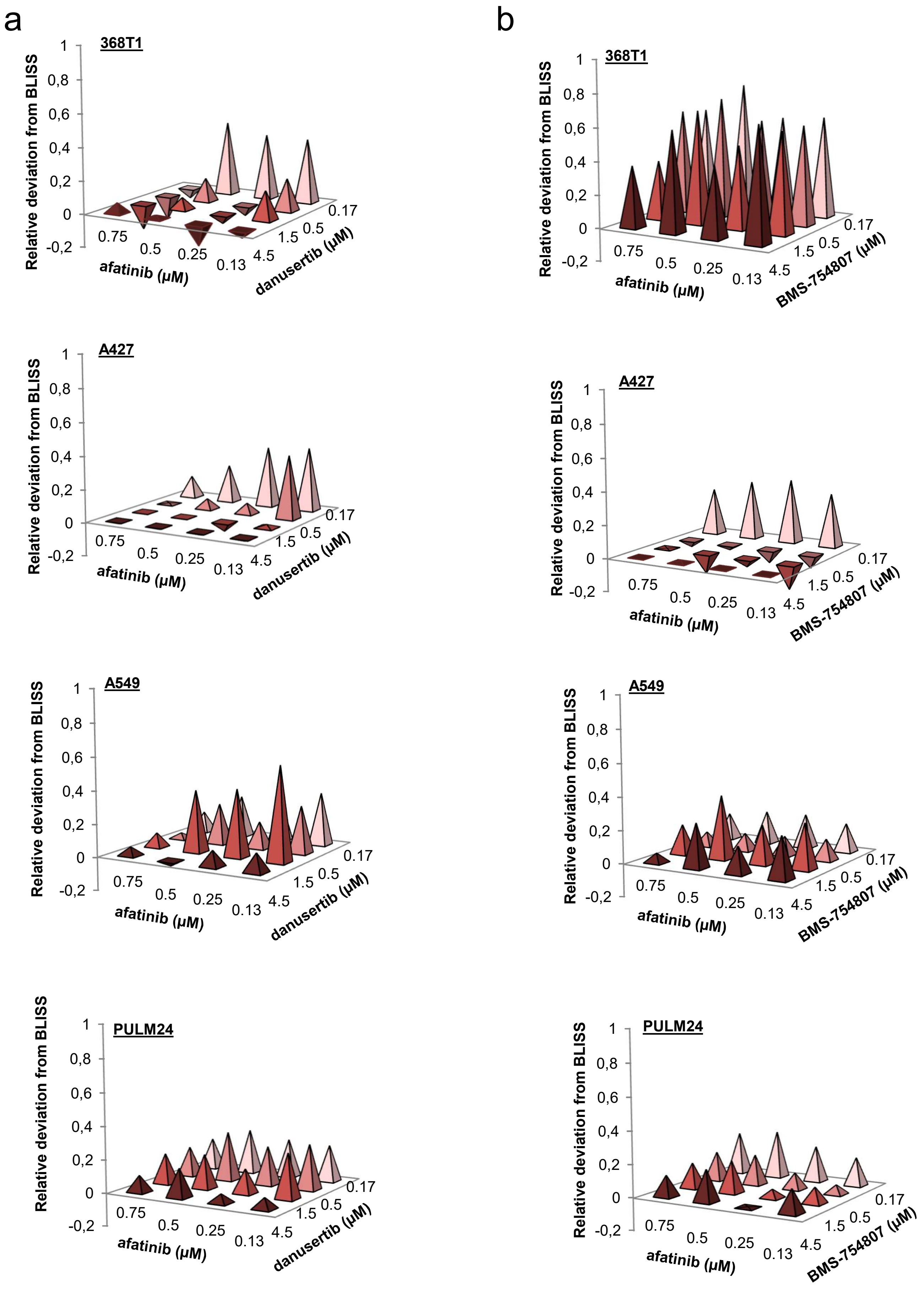
a
368T1
Relative deviation from BLISS
1
0,8
0,6
0,4
0,2
0
-0,2
0.75
0.5
0.25
0.13
afatinib (µM)
4.5
1.5
0.5
0.17
danusertib (µM)
A427
Relative deviation from BLISS
afatinib (µM)
danusertib (µM)
A549
Relative deviation from BLISS
afatinib (µM)
danusertib (µM)
PULM24
Relative deviation from BLISS
afatinib (µM)
danusertib (µM)
b
368T1
Relative deviation from BLISS
afatinib (µM)
BMS-754807 (µM)
A427
Relative deviation from BLISS
afatinib (µM)
BMS-754807 (µM)
A549
Relative deviation from BLISS
afatinib (µM)
BMS-754807 (µM)
PULM24
Relative deviation from BLISS
afatinib (µM)
BMS-754807 (µM)

a

AURKA

	Min	Q1	Med	Q3	Max	Upper whisker	N
Normal	20	45	65	94	424	149	57
Tumor	51	189	450	696	1875	1423	57

b

AURKB

	Min	Q1	Med	Q3	Max	Upper whisker	N
Normal	2	12	18	37	119	66	57
Tumor	16	82	157	405	1123	886	57

a

	PULM21	PULM24	A549	MML416	MML884	KP
**Origin**	Human	Human	Human	Murine	Murine	Murine
**NSCLC histology**	Adeno-carcinoma	Adeno-carcinoma	Adeno-carcinoma	Adeno-carcinoma	Adeno-carcinoma	Adeno-carcinoma
KRAS	G12C	G12C	G12S	G12C	G12C	G12D
p53	null	null	wild-type	null	null	null

b

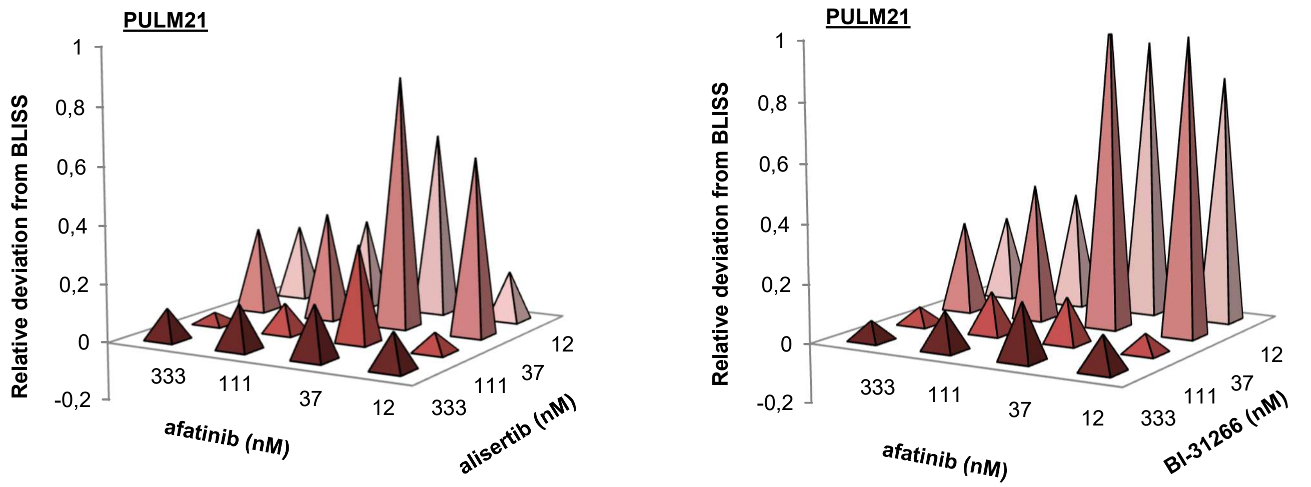

c

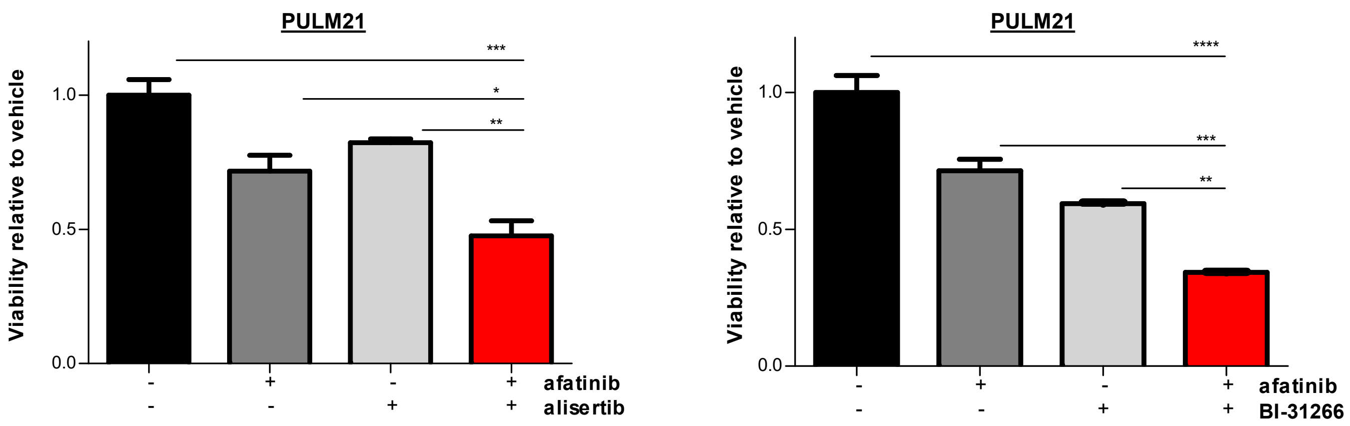

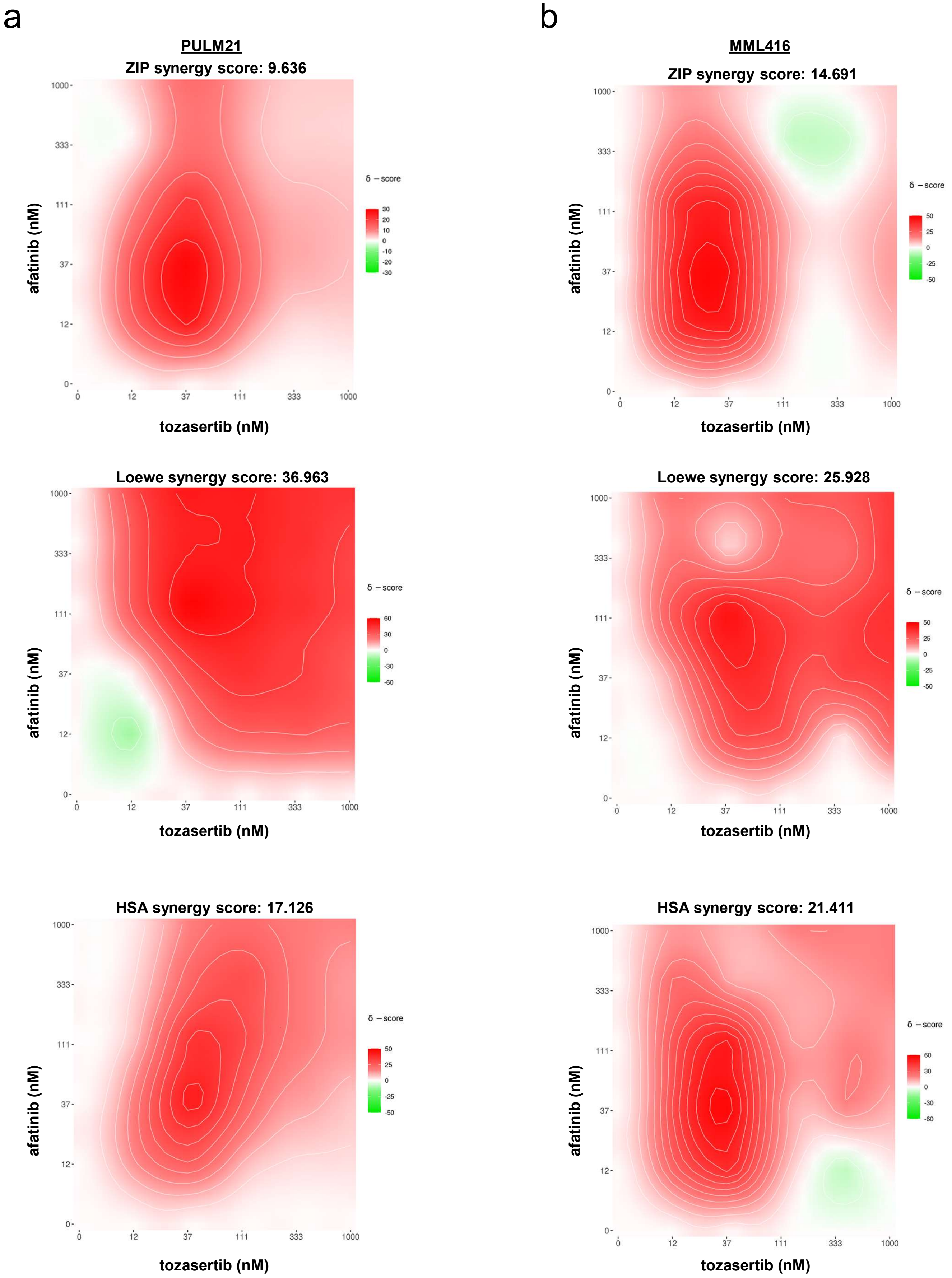
a
PULM21
ZIP synergy score: 9.636
Loewe synergy score: 36.963
HSA synergy score: 17.126
b
MML416
ZIP synergy score: 14.691
Loewe synergy score: 25.928
HSA synergy score: 21.411
δ – score
afatinib (nM)
tozasertib (nM)
0
12
37
111
333
1000

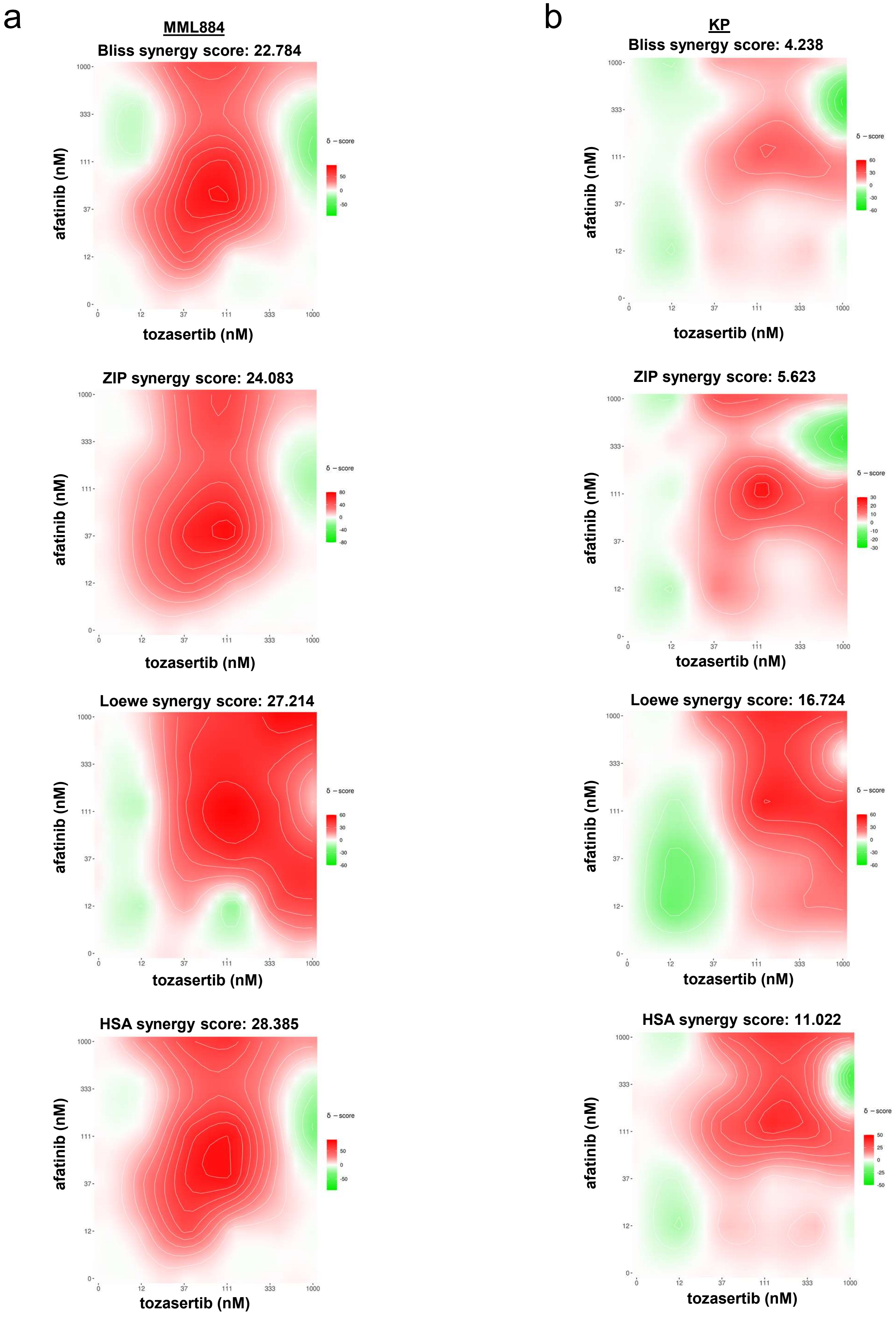
a
MML884
Bliss synergy score: 22.784
ZIP synergy score: 24.083
Loewe synergy score: 27.214
HSA synergy score: 28.385
b
KP
Bliss synergy score: 4.238
ZIP synergy score: 5.623
Loewe synergy score: 16.724
HSA synergy score: 11.022
afatinib (nM)
tozasertib (nM)
δ – score
0
12
37
111
333
1000

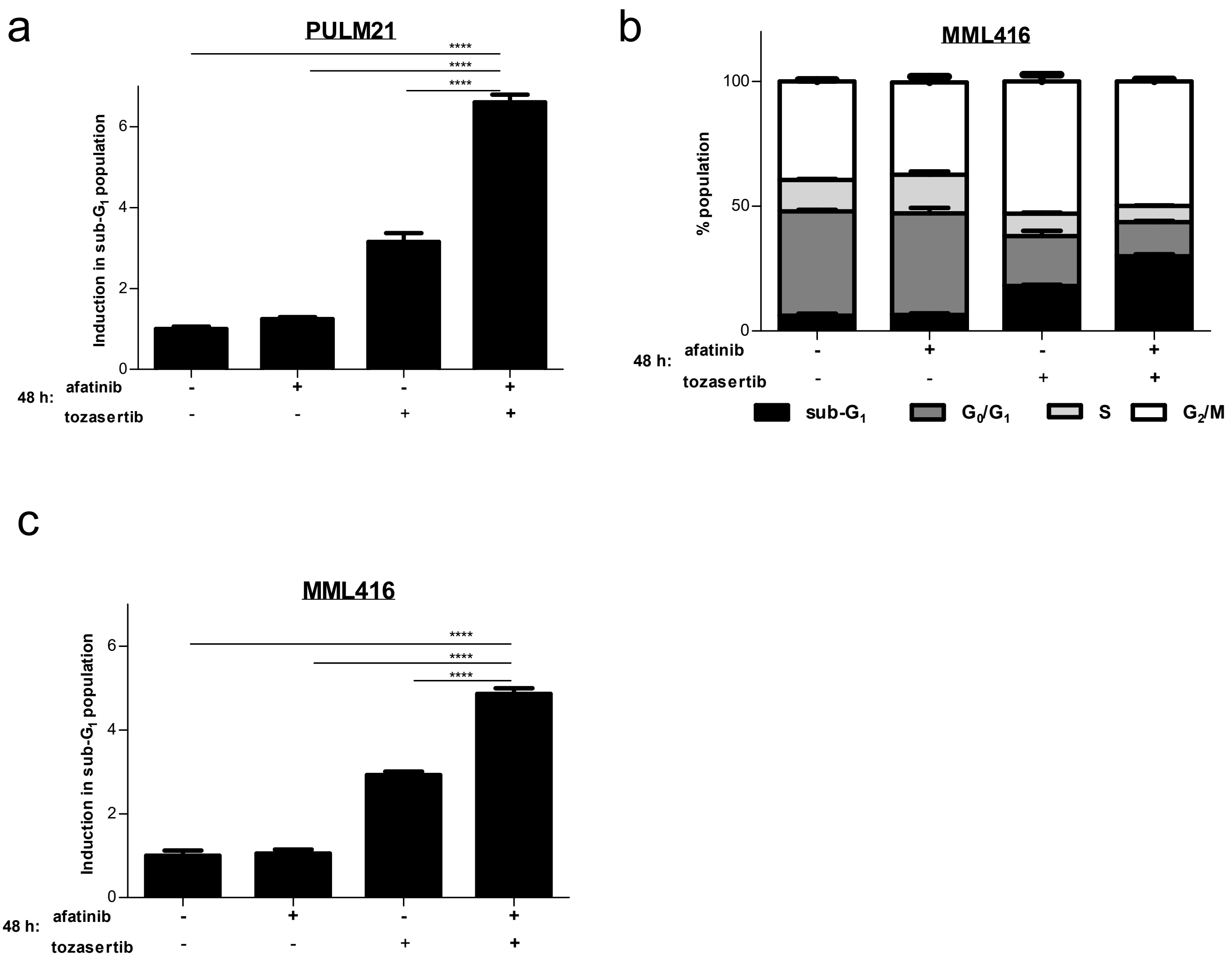
a
PULM21
****
****
****
Induction in sub-G1 population
0
2
4
6
48 h:
afatinib
tozasertib
b
MML416
% population
0
50
100
48 h:
afatinib
tozasertib
sub-G1
G0/G1
S
G2/M
c
MML416
****
****
****
Induction in sub-G1 population
0
2
4
6
48 h:
afatinib
tozasertib

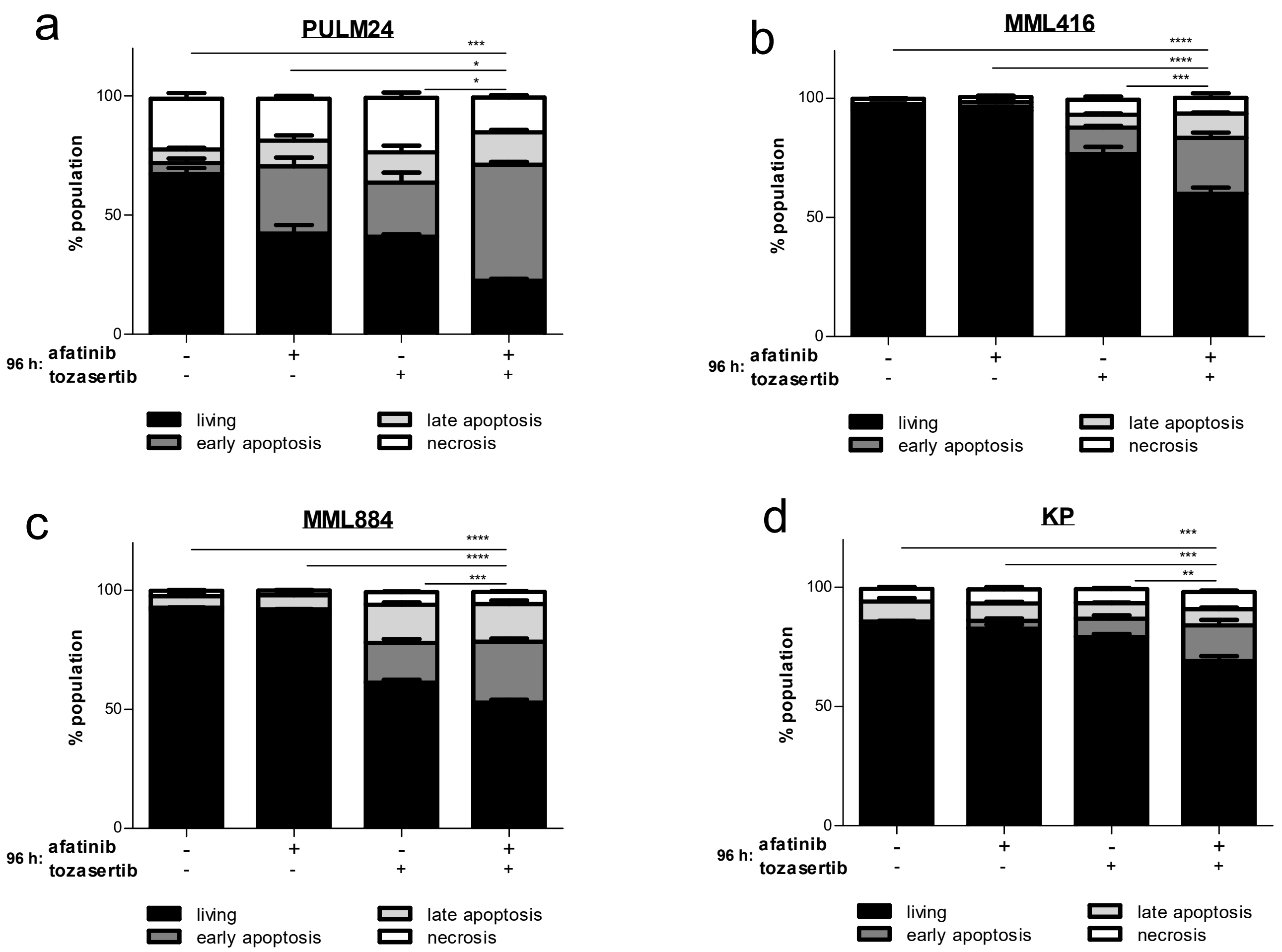

a
PULM24
b
MML416
c
MML884
d
KP
% population
96 h:
afatinib
tozasertib
living
early apoptosis
late apoptosis
necrosis

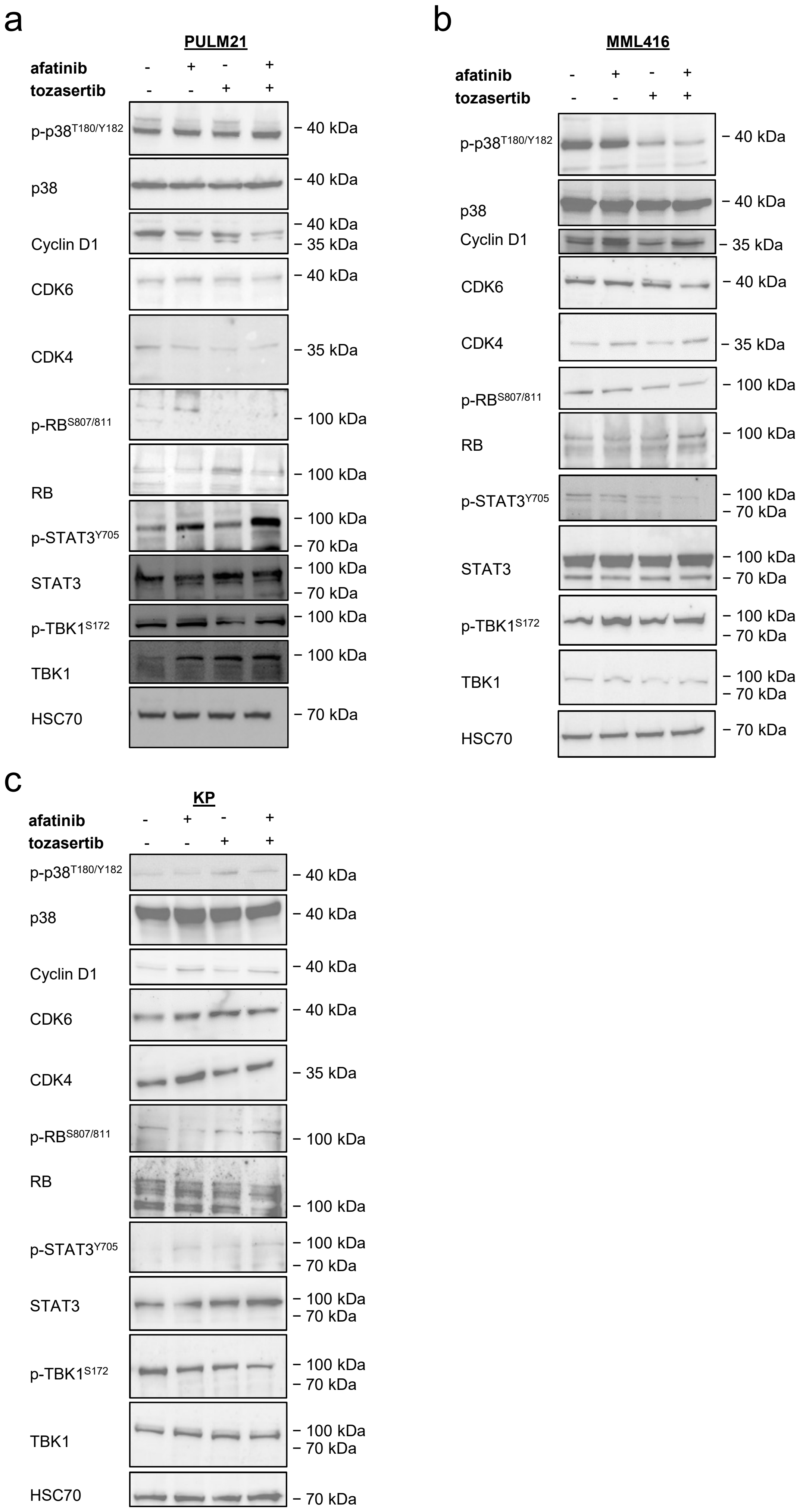

a
PULM21
afatinib - + - +
tozasertib - - + +
p-p38T180/Y182
p38
Cyclin D1
CDK6
CDK4
p-RBS807/811
RB
p-STAT3Y705
STAT3
p-TBK1S172
TBK1
HSC70
– 40 kDa
– 40 kDa
– 40 kDa
– 35 kDa
– 40 kDa
– 35 kDa
– 100 kDa
– 100 kDa
– 100 kDa
– 70 kDa
– 100 kDa
– 70 kDa
– 100 kDa
– 100 kDa
– 70 kDa
b
MML416
afatinib - + - +
tozasertib - - + +
p-p38T180/Y182
p38
Cyclin D1
CDK6
CDK4
p-RBS807/811
RB
p-STAT3Y705
STAT3
p-TBK1S172
TBK1
HSC70
– 40 kDa
– 40 kDa
– 35 kDa
– 40 kDa
– 35 kDa
– 100 kDa
– 100 kDa
– 100 kDa
– 70 kDa
– 100 kDa
– 70 kDa
– 100 kDa
– 70 kDa
– 100 kDa
– 70 kDa
– 70 kDa
c
KP
afatinib - + - +
tozasertib - - + +
p-p38T180/Y182
p38
Cyclin D1
CDK6
CDK4
p-RBS807/811
RB
p-STAT3Y705
STAT3
p-TBK1S172
TBK1
HSC70
– 40 kDa
– 40 kDa
– 40 kDa
– 40 kDa
– 35 kDa
– 100 kDa
– 100 kDa
– 100 kDa
– 70 kDa
– 100 kDa
– 70 kDa
– 100 kDa
– 70 kDa
– 100 kDa
– 70 kDa
– 70 kDa

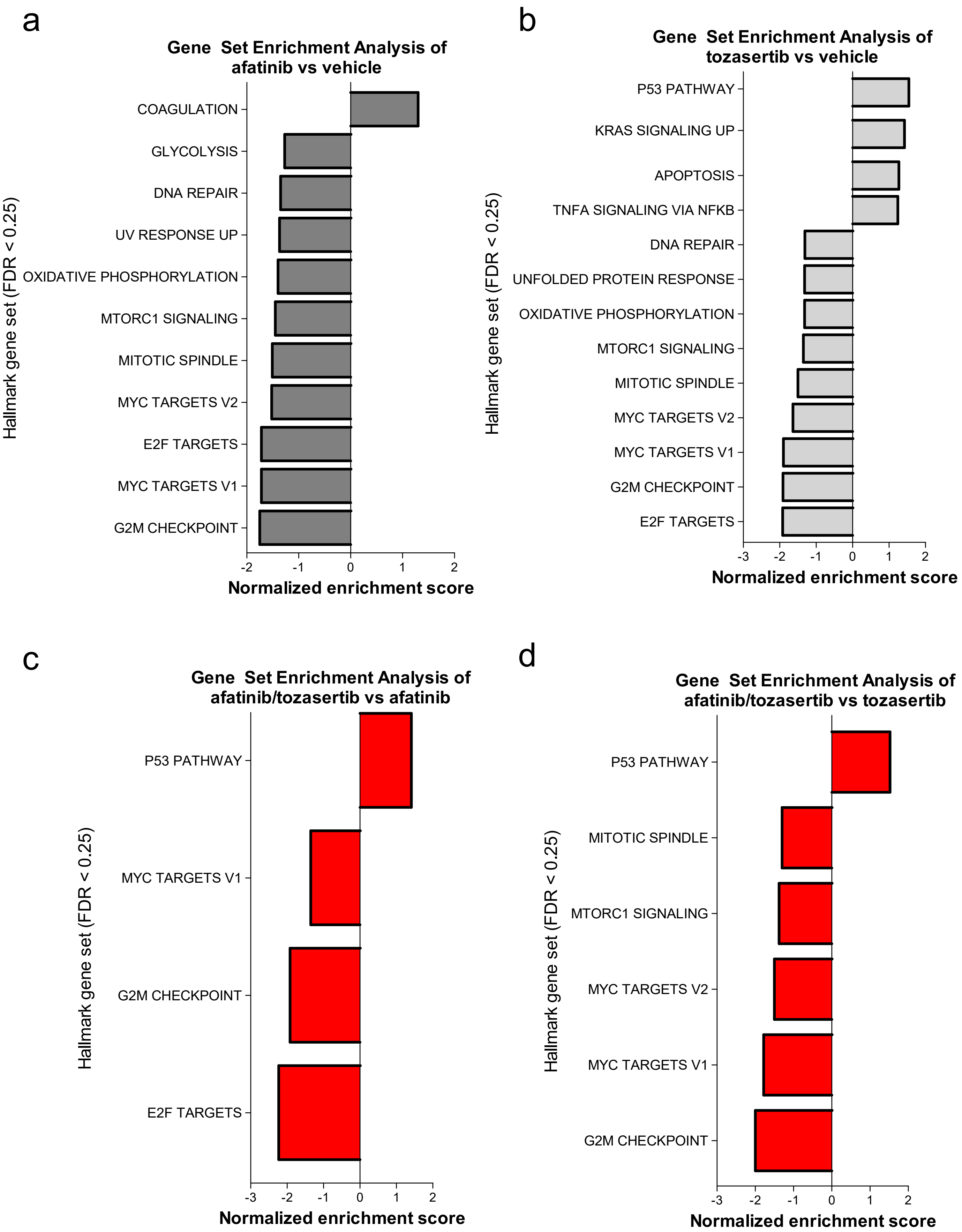
a
Gene Set Enrichment Analysis of
afatinib vs vehicle
COAGULATION
GLYCOLYSIS
DNA REPAIR
UV RESPONSE UP
OXIDATIVE PHOSPHORYLATION
MTORC1 SIGNALING
MITOTIC SPINDLE
MYC TARGETS V2
E2F TARGETS
MYC TARGETS V1
G2M CHECKPOINT
Hallmark gene set (FDR < 0.25)
-2
-1
0
1
2
Normalized enrichment score
b
Gene Set Enrichment Analysis of
tozasertib vs vehicle
P53 PATHWAY
KRAS SIGNALING UP
APOPTOSIS
TNFA SIGNALING VIA NFKB
DNA REPAIR
UNFOLDED PROTEIN RESPONSE
OXIDATIVE PHOSPHORYLATION
MTORC1 SIGNALING
MITOTIC SPINDLE
MYC TARGETS V2
MYC TARGETS V1
G2M CHECKPOINT
E2F TARGETS
Hallmark gene set (FDR < 0.25)
-3
-2
-1
0
1
2
Normalized enrichment score
c
Gene Set Enrichment Analysis of
afatinib/tozasertib vs afatinib
P53 PATHWAY
MYC TARGETS V1
G2M CHECKPOINT
E2F TARGETS
Hallmark gene set (FDR < 0.25)
-3
-2
-1
0
1
2
Normalized enrichment score
d
Gene Set Enrichment Analysis of
afatinib/tozasertib vs tozasertib
P53 PATHWAY
MITOTIC SPINDLE
MTORC1 SIGNALING
MYC TARGETS V2
MYC TARGETS V1
G2M CHECKPOINT
Hallmark gene set (FDR < 0.25)
-3
-2
-1
0
1
2
Normalized enrichment score

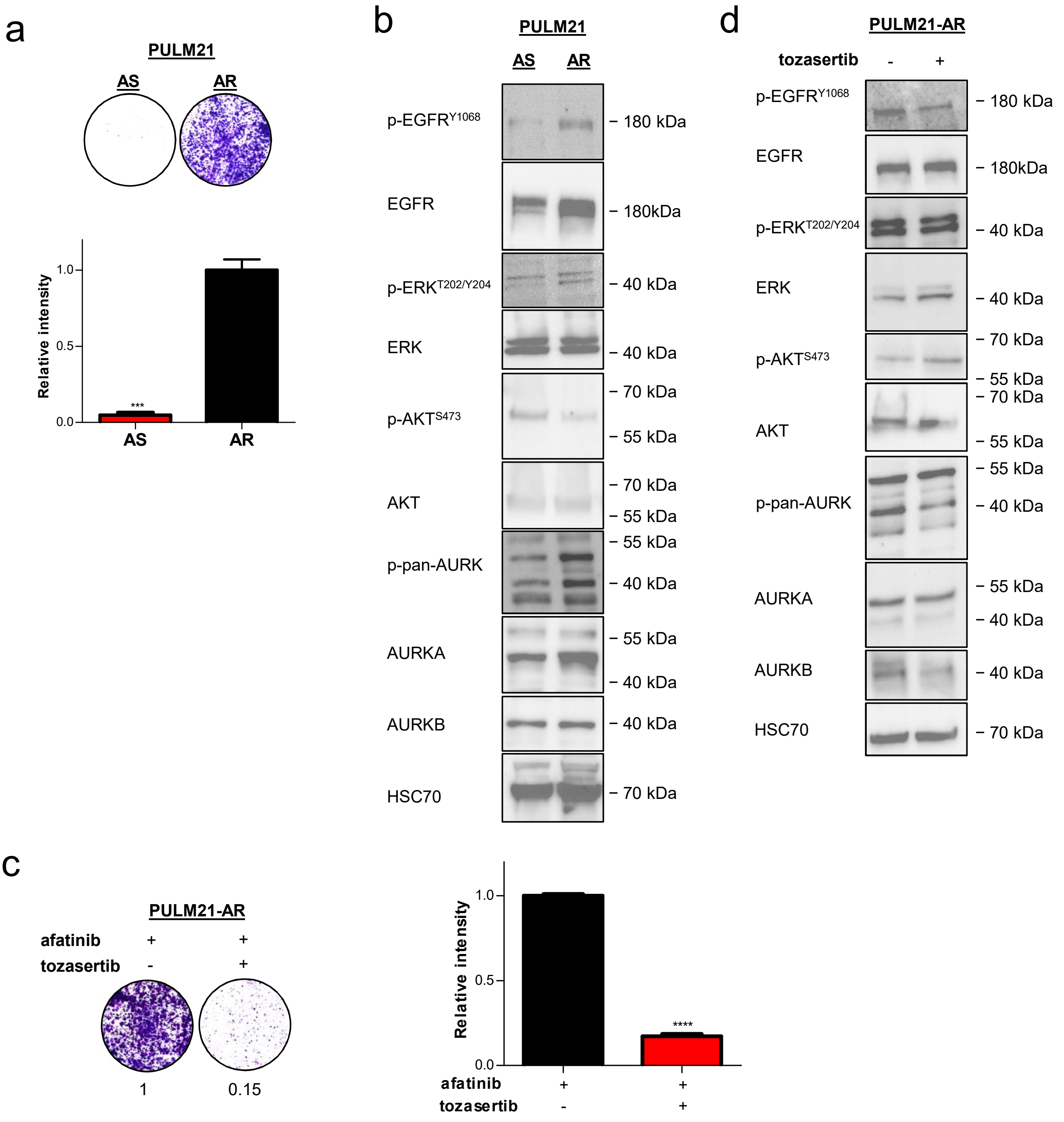
a
PULM21
AS
AR
Relative intensity
1.0
0.5
0.0
***
AS
AR
b
PULM21
AS
AR
p-EGFR Y1068
EGFR
p-ERK T202/Y204
ERK
p-AKT S473
AKT
p-pan-AURK
AURKA
AURKB
HSC70
180 kDa
180kDa
40 kDa
40 kDa
70 kDa
55 kDa
70 kDa
55 kDa
55 kDa
40 kDa
55 kDa
40 kDa
40 kDa
70 kDa
c
PULM21-AR
afatinib + +
tozasertib - +
1
0.15
Relative intensity
1.0
0.5
0.0
****
afatinib + +
tozasertib - +
d
PULM21-AR
tozasertib - +
p-EGFR Y1068
EGFR
p-ERK T202/Y204
ERK
p-AKT S473
AKT
p-pan-AURK
AURKA
AURKB
HSC70
180 kDa
180kDa
40 kDa
40 kDa
70 kDa
55 kDa
70 kDa
55 kDa
55 kDa
40 kDa
55 kDa
40 kDa
40 kDa
70 kDa

Figure S1. Overview of the compounds used in the high-throughput drug screening in *KRAS* mutated LUAD cells. a Summary of inhibitors tested. **b** Agents that enhance afatinib-induced growth inhibition in both 368T1 and PULM24 cell lines.

Figure S2. Overview of growth inhibition by various pathway inhibitors ± afatinib. a Viability of 368T1 cells upon inhibitor alone (10-50 µM) or in combination with afatinib (1 µM) was normalized against that by the vehicle DMSO (set to 1). Cell viability was assessed by an ATP-dependent luminescent assay at 72 h. Each circle represents one compound. * $P < 0.05$, ** $P < 0.01$, *** $P < 0.001$, **** $P < 0.0001$ (Student's *t* test). **b** Viability of PULM24 cells upon inhibitor alone (10-50 µM) or in combination with afatinib (1 µM) was normalized against that by the vehicle DMSO (set to 1). Cell viability was assessed by an ATP-dependent luminescent assay at 72 h. Each circle represents one compound. * $P < 0.05$, ** $P < 0.01$, *** $P < 0.001$, **** $P < 0.0001$, n.s., not significant (Student's *t* test).

Figure S3. Overview of the agents used in pairwise drug combination viability assays. a Characteristics of NSCLC adenocarcinoma cell lines used in the 6x6 matrices. **b** Compound hits identified across a panel of LUAD cells. +: potential synergy; -: lack of synergy between afatinib and indicated compound.

Figure S4. Pairwise drug combination viability screen reveals potential synergistic partners of afatinib. a Heat maps, depicting variation in drug interactions, are shown for the combination of afatinib with the pan-AURK inhibitor danusertib in the indicated *KRAS* mutated LUAD cell lines. Co-treatments were tested with afatinib in 6x6 matrices. Each matrix included a dose-response of each drug alone together with solvent (DMSO) only; thus a 6x6 matrix included five doses each tested in combination (36 combinations in total). Following a 3 day incubation cell viability was measured by the CellTiter-Glo (CTG) Viability Assay. Blue, dead; red, viable. **b** Heat maps, depicting variation in drug interactions, are shown for the combination of afatinib with the IGF1R inhibitor BMS-754807 in the indicated *KRAS* mutated LUAD cell lines. Co-treatments were tested with afatinib in 6x6 matrices. Each matrix included a dose-response of each drug alone together with DMSO only; a 6x6 matrix included five doses each tested in combination (36 combinations in total). Following a 3 day incubation cell viability was measured by the CTG Viability Assay. Blue, dead; red, viable.

Figure S5. Bliss prediction model of afatinib in combination with inhibitors of Aurora kinase and IGF1R. a Combined effect of afatinib with the Aurora kinase inhibitor danusertib exceeds Bliss prediction indicating synergy at low nanomolar concentrations. Analysis was carried out in triplicate. Values depicted represent absolute deviations. Observed values are divided through standard deviations (SDs) plus 15th percentile. Needle graphs indicate deviation from Bliss predicted synergy in *KRAS* mutated LUAD cells (murine: 368T1; human: A427, A459, PULM24). **b** Combined effects of afatinib with the IGF1R inhibitor BMS-754807

exceeds Bliss prediction indicating synergy at low nanomolar concentrations. Analysis was carried out in triplicate. Values depicted represent absolute deviations. Observed values are divided through standard deviations (SDs) plus 15th percentile. Needle graphs indicate deviation from Bliss predicted synergy in *KRAS* mutated LUAD cells (murine: 368T1; human: A427, A459, PULM24).

Figure S6. Levels of *AURKA* and *AURKB* are elevated in lung adenocarcinoma. a Relative *AURKA* mRNA expression in paired lung adenocarcinoma and adjacent normal tissues (TNMplot.com)[1,2]. Fold change median (*AURKA*): 6.92; n=57; **** $P< 0.0001$ (Mann-Whitney test). **b** Relative *AURKB* mRNA expression in paired lung adenocarcinoma and adjacent normal tissues (TNMplot.com)[1,2]. Fold change median (*AURKB*): 8.72; n=57; **** $P< 0.0001$ (Mann-Whitney test).

Figure S7. Co-inhibition of ERBB with AURKA or AURKB suppresses the viability of *KRAS* mutated LUAD cells. a Characteristics of NSCLC adenocarcinoma cell lines used in the follow-up experiments. **b** Alisertib (an Aurora kinase A inhibitor) and BI-31266 (an Aurora kinase B inhibitor) were tested with afatinib in 6x6 matrices. Viability was measured after 96 hours by the CTG Viability Assay. Combined effects of afatinib with different Aurora kinase inhibitors exceed Bliss prediction indicating synergy. **c** PULM21 cells were treated with vehicle or the indicated compounds (afatinib: 111 nM; alisertib: 37 nM; BI-31266: 37 nM) for 96 hours. Cell viability and proliferation were assessed using the CTG assay. Error bars indicate ±SEM. Analysis was performed in triplicate. * $P< 0.05$, ** $P< 0.01$, *** $P< 0.001$, **** $P< 0.0001$, n.s., not significant (One-way ANOVA with subsequent Bonferroni posttest).

Figure S8. Synergy score analysis of combined ERBB and AURK inhibition in PULM21 and MML416 cells. a Pairwise drug combination viability assay was performed using PULM21 cells. Indicated compounds were tested in 6x6 matrices. Each matrix included a dose-response of each drug alone together with DMSO only; a 6x6 matrix included five doses each tested in combination (36 combinations in total). Following a 4 day incubation cell viability was measured by the CTG Viability Assay. Synergy scoring was determined using multiple reference models. Visualisation was done using SynergyFinder 3.0 online tool. **b** Pairwise drug combination viability assay was performed using MML416 cells. Indicated compounds were tested in 6x6 matrices. Each matrix included a dose-response of each drug alone together with DMSO only; a 6x6 matrix included five doses each tested in combination (36 combinations in total). Following a 4 day incubation cell viability was measured by the CTG Viability Assay. Synergy scoring was determined using multiple reference models. Visualisation was done using SynergyFinder 3.0 online tool.

Figure S9. Synergy score analysis of combined ERBB and AURK inhibition in MML884 and KP cells. a Pairwise drug combination viability assay was performed using MML884 cells.

Indicated compounds were tested in 6x6 matrices. Each matrix included a dose-response of each drug alone together with DMSO only; a 6x6 matrix included five doses each tested in combination (36 combinations in total). Following a 4 day incubation cell viability was measured by the CTG Viability Assay. Synergy scoring was determined using multiple reference models. Visualisation was done using SynergyFinder 3.0 online tool. **b** Pairwise drug combination viability assay was performed using KP cells. Indicated compounds were tested in 6x6 matrices. Each matrix included a dose-response of each drug alone together with DMSO only; a 6x6 matrix included five doses each tested in combination (36 combinations in total). Following a 4 day incubation cell viability was measured by the CTG Viability Assay. Synergy scoring was determined using multiple reference models. Visualisation was done using SynergyFinder 3.0 online tool.

Figure S10. Combined ERBB and AURK inhibition induces cell cycle arrest in *KRAS* mutant cells. a PULM21 cells were incubated with vehicle, single agent (37 nM), or the combination for 48 hours, stained with PI, and analyzed by flow cytometry. Combination treatment resulted in a pronounced accumulation in sub-G_1 fraction compared to single agents. Analysis was carried out in triplicate. Data are presented as mean values ±SEM. **** $P< 0.0001$ (One-way ANOVA with subsequent Bonferroni posttest). **b** MML416 cells were incubated with vehicle, single agent (111 nM), or the combination for 48 hours, stained with PI, and analyzed by flow cytometry. Combination treatment resulted in enhanced cell cycle arrest. Analysis was carried out in triplicate. Data are presented as mean values ±SEM. **c** MML416 cells were incubated with vehicle, single agent (111 nM), or the combination for 48 hours, stained with PI, and analyzed by flow cytometry. Combination treatment resulted in an accumulation in sub-G_1 fraction. Analysis was carried out in triplicate. Data are presented as mean values ±SEM. **** $P< 0.0001$ (One-way ANOVA with subsequent Bonferroni posttest).

Figure S11. AURK inhibition enhances afatinib-induced apoptosis in LUAD cells with *KRAS* mutations. a PULM24 cells were incubated with vehicle, single agent (37 nM each compound), or the combination for 96 hours, stained with Annexin V/7-AAD, and analyzed by flow cytometry. Analysis was carried out in triplicate. Data are presented as mean values ±SEM. * $P< 0.05$, *** $P< 0.001$ (One-way ANOVA with subsequent Bonferroni posttest). **b** MML416 cells were incubated with vehicle, single agent (111 nM each compound), or the combination for 96 hours, stained with Annexin V/7-AAD, and analyzed by flow cytometry. Analysis was carried out in triplicate. Data are presented as mean values ±SEM. *** $P< 0.001$, **** $P< 0.0001$ (One-way ANOVA with subsequent Bonferroni posttest). **c** MML884 cells were incubated with vehicle, single agent (111 nM each compound), or the combination for 96 hours, stained with Annexin V/7-AAD, and analyzed by flow cytometry. Analysis was carried out in triplicate. Data are presented as mean values ±SEM. *** $P< 0.001$, **** $P< 0.0001$ (One-way

ANOVA with subsequent Bonferroni posttest). **d** KP cells were incubated with vehicle, single agent (111 nM each compound), or the combination for 96 hours, stained with Annexin V/7-AAD, and analyzed by flow cytometry. Analysis was carried out in triplicate. Data are presented as mean values ±SEM. ** *P*< 0.01, *** *P*< 0.001 (One-way ANOVA with subsequent Bonferroni posttest).

Figure S12. Signaling analysis upon afatinib/tozasertib co-treatment in *KRAS* mutated LUAD cells. a PULM21 cells treated with the indicated agents (37 nM each compound) for 96 hours were assessed by immunoblots. HSC70 served as a loading control. **b** MML416 cells treated with the indicated agents (111 nM each compound) for 96 hours were assessed by immunoblots. HSC70 served as a loading control. **c** KP cells treated with the indicated agents (111 nM each compound) for 96 hours were assessed by immunoblots. HSC70 served as a loading control.

Figure S13. Comparative gene set enrichment analysis upon afatinib and tozasertib exposure in PULM21 cells. a Overview of gene set enrichment analysis of afatinib vs vehicle treatment. The Hallmark Gene Set Collection was used as a reference. False discovery rate <0.25 was considered significant (NOM *P*< 0.05). **b** Overview of gene set enrichment analysis of tozasertib vs vehicle treatment. The Hallmark Gene Set Collection was used as a reference. False discovery rate <0.25 was considered significant (NOM *P*< 0.05). **c** Overview of gene set enrichment analysis comparing afatinib/tozasertib co-treatment vs afatinib alone. The Hallmark Gene Set Collection was used as a reference. False discovery rate <0.25 was considered significant (NOM *P*< 0.05). **d** Overview of gene set enrichment analysis comparing afatinib/tozasertib co-treatment vs tozasertib single agent. The Hallmark Gene Set Collection was used as a reference. False discovery rate <0.25 was considered significant (NOM *P*< 0.05).

Figure S14. Afatinib-resistant *KRAS* mutated cells remain sensitive to AURK inhibition. a Exposure to increasing concentrations of afatinib over time conferred PULM21 cells resistant to the treatment (1 µM). Representative images of crystal violet staining of colonies after ten days are shown (n=3 per group). ImageJ was used for quantification. Data are presented as mean values ±SEM. *** *P*< 0.001 (Student's *t* test). Sensitive cells are indicated as AS; resistant cells are labeled with AR. **b** Changes in signaling upon resistance to afatinib were assessed by immunoblot. HSC70 was used as a loading control. **c** PULM21-AR cells were treated with vehicle or the indicated drugs (37 nM each compound). Representative images of crystal violet staining of colonies after ten days are shown (n=3 per group). ImageJ was used for quantification. Error bars indicate ± SEM. **** *P*< 0.0001 (Student's *t* test). **d** PULM21-AR were treated with vehicle or tozasertib (37 nM) and assessed by immunoblot at 48 hours. HSC70 served as a loading control.

Supplementary References

1. Bartha, Á. & Győrffy, B. TNMplot.com: A Web Tool for the Comparison of Gene Expression in Normal, Tumor and Metastatic Tissues. *Int. J. Mol. Sci.* **22**, 2622 (2021).
2. Györffy, B. Transcriptome-level discovery of survival-associated biomarkers and therapy targets in non-small-cell lung cancer. *Br J Pharmacol.* **181**, 362–374 (2023).

Western Blot original data

Source data for Figure 5b

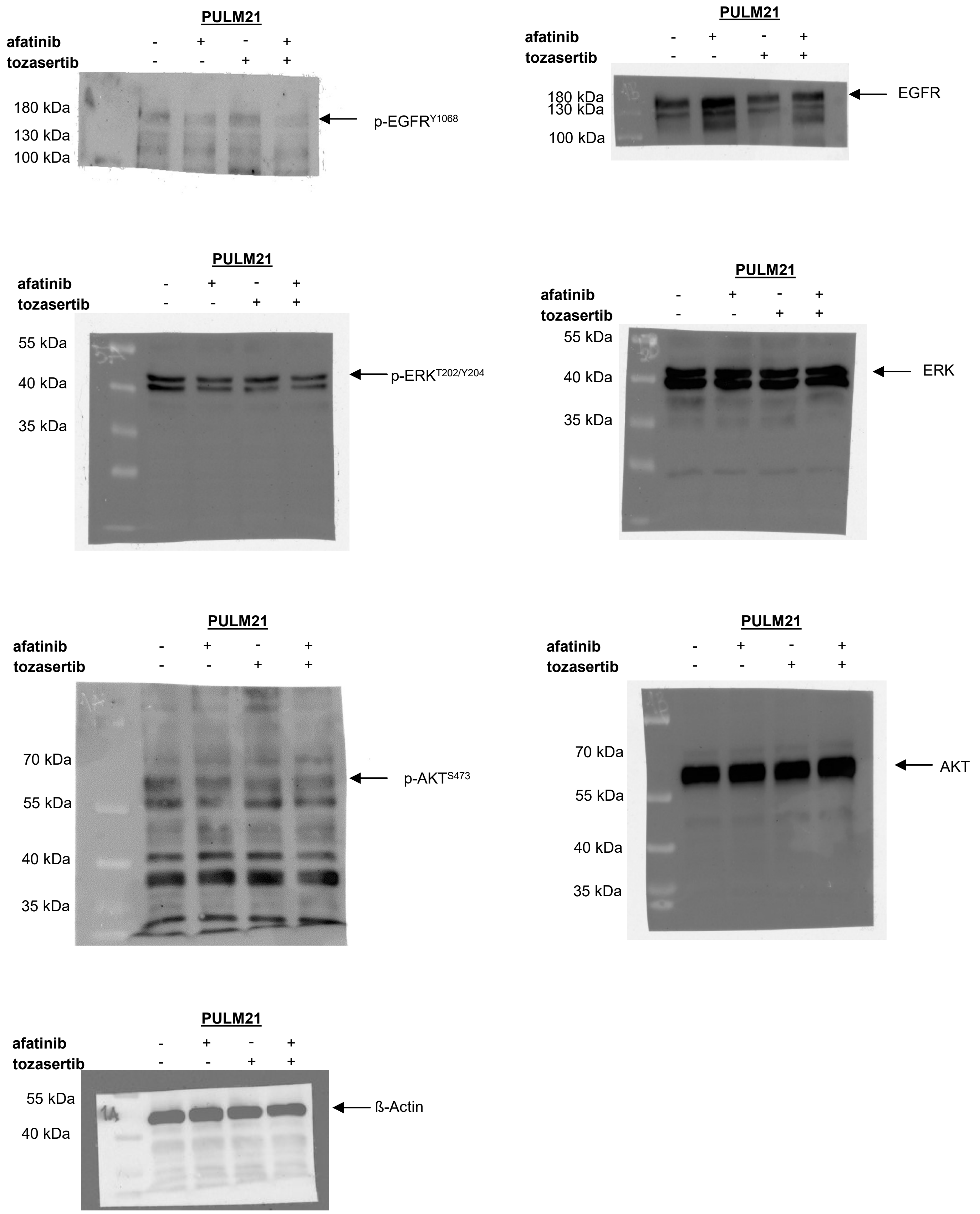

Source data for Figure 5b

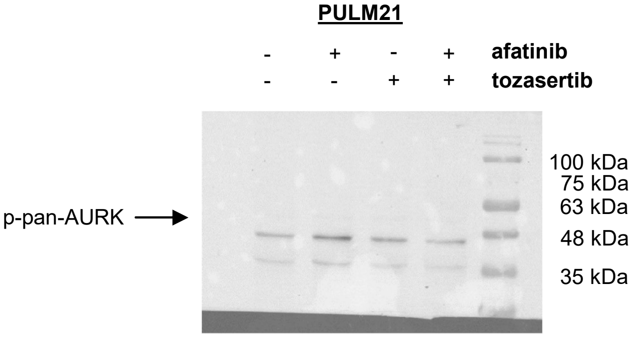

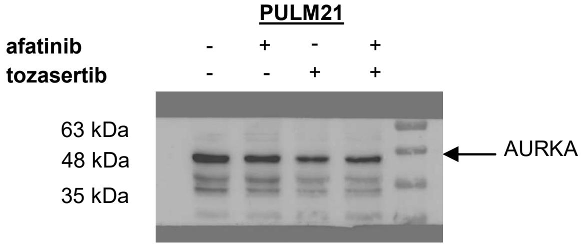

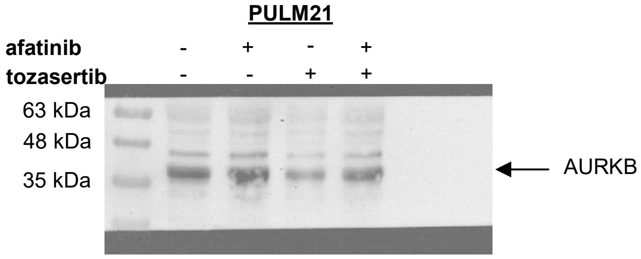

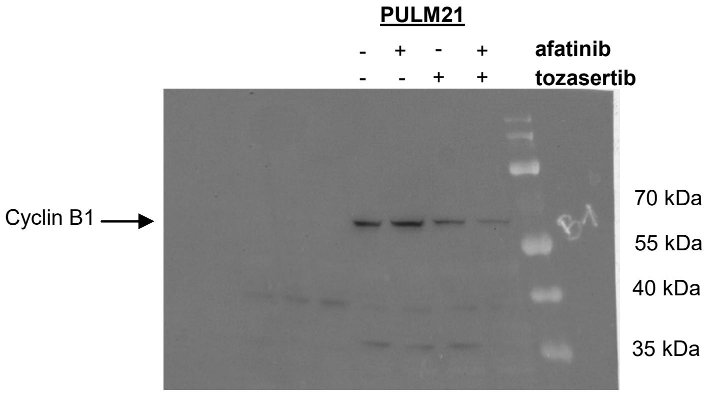

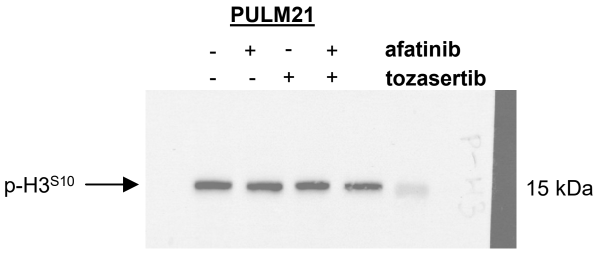

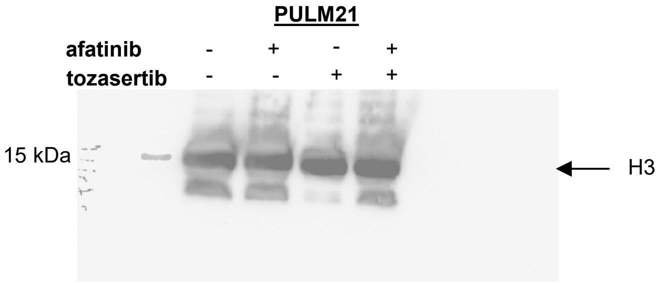

Source data for Figure 5b

MML416

afatinib - + - +
tozasertib - - + +

180 kDa
100 kDa

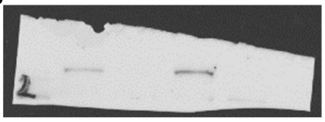

p-EGFR^{Y1068}

MML416

afatinib - + - +
tozasertib - - + +

180 kDa
130 kDa
100 kDa

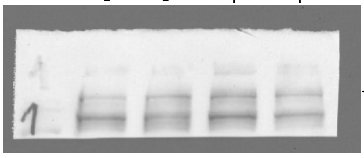

EGFR

MML416

afatinib - + - +
tozasertib - - + +

55 kDa
40 kDa
35 kDa

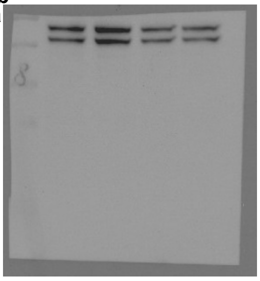

p-ERK$^{T202/Y204}$

MML416

afatinib - + - +
tozasertib - - + +

55 kDa
40 kDa
35 kDa

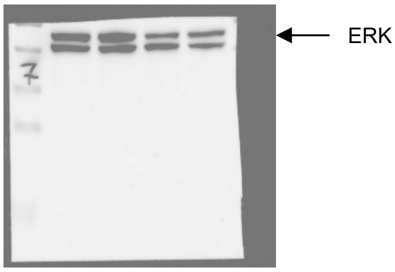

ERK

MML416

afatinib - + - +
tozasertib - - + +

70 kDa
55 kDa
40 kDa

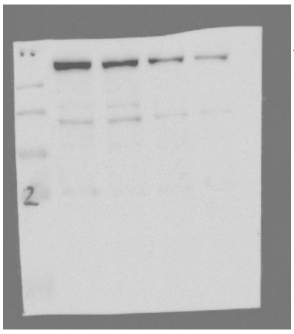

p-AKT^{S473}

MML416

afatinib - + - +
tozasertib - - + +

70 kDa
55 kDa

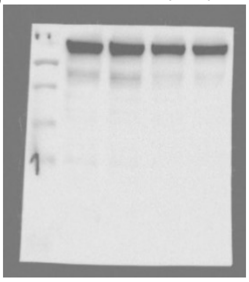

AKT

MML416

afatinib - + - +
tozasertib - - + +

100 kDa
70 kDa

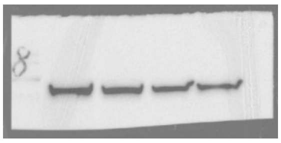

HSC70

Source data for Figure 5b

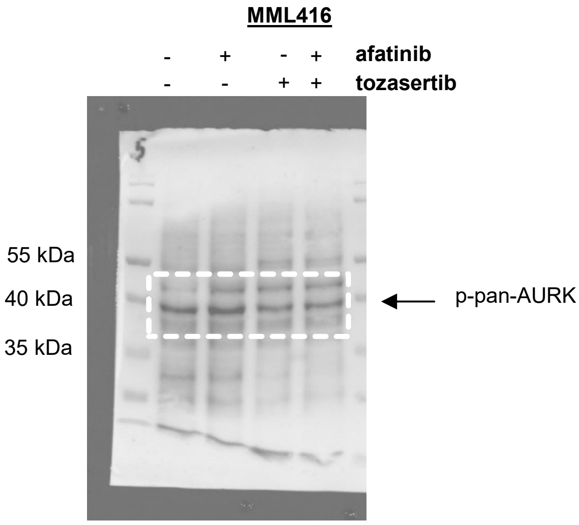

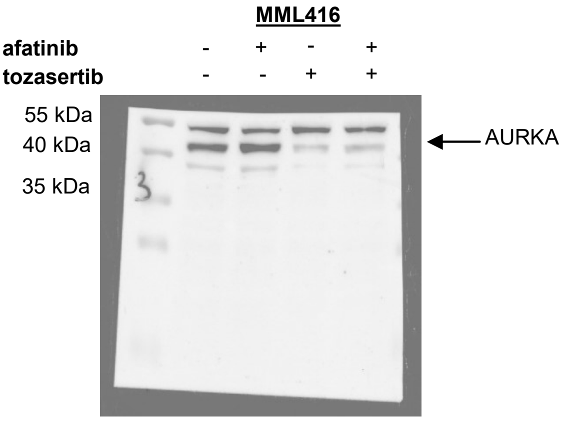

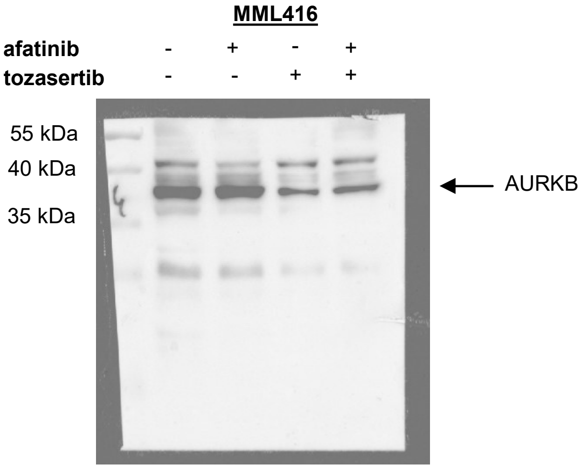

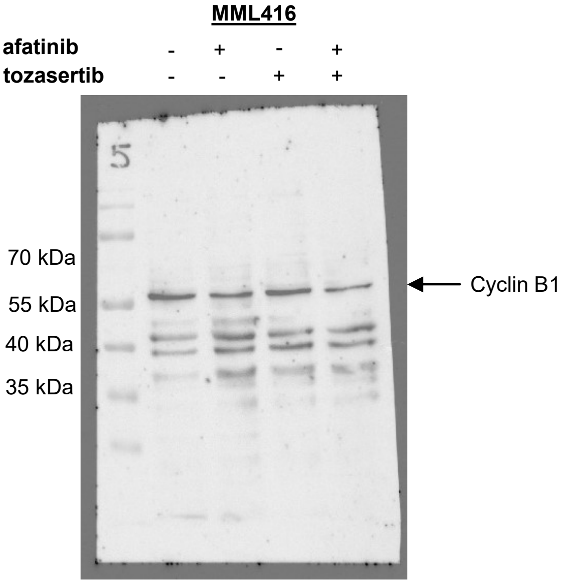

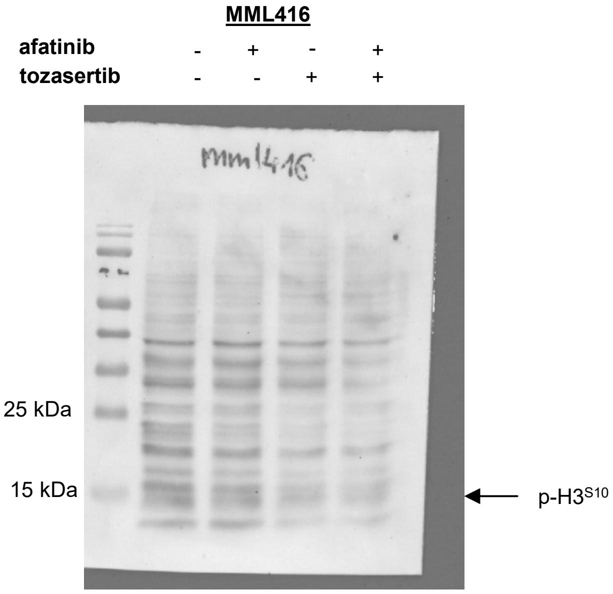

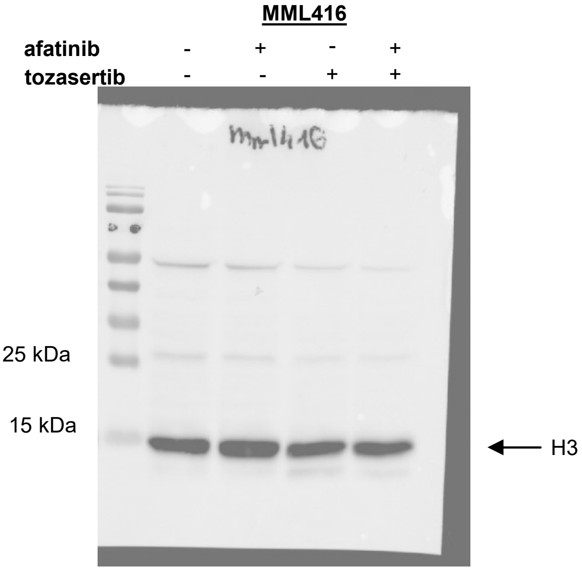

Source data for Figure 5b

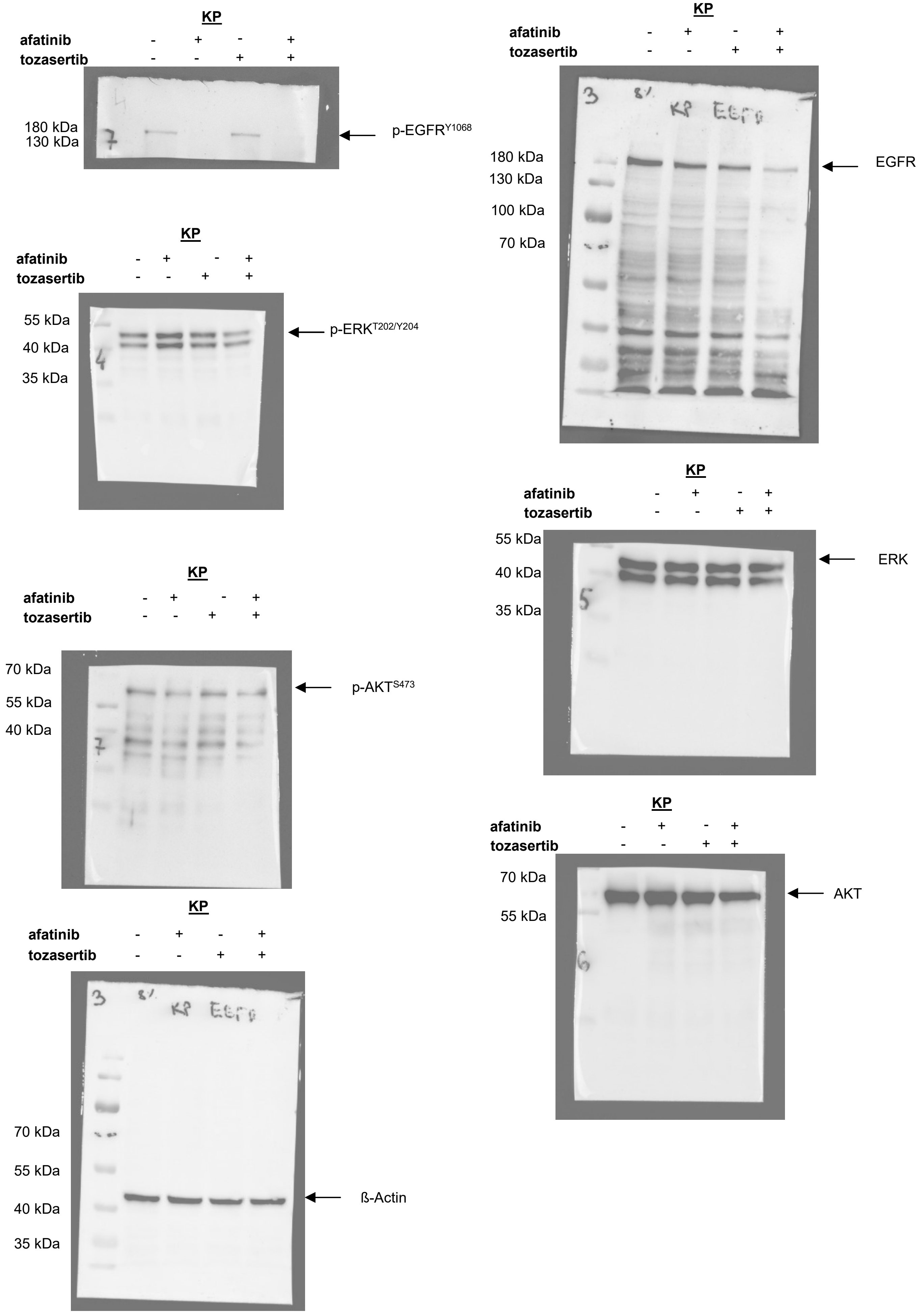

Source data for Figure 5b

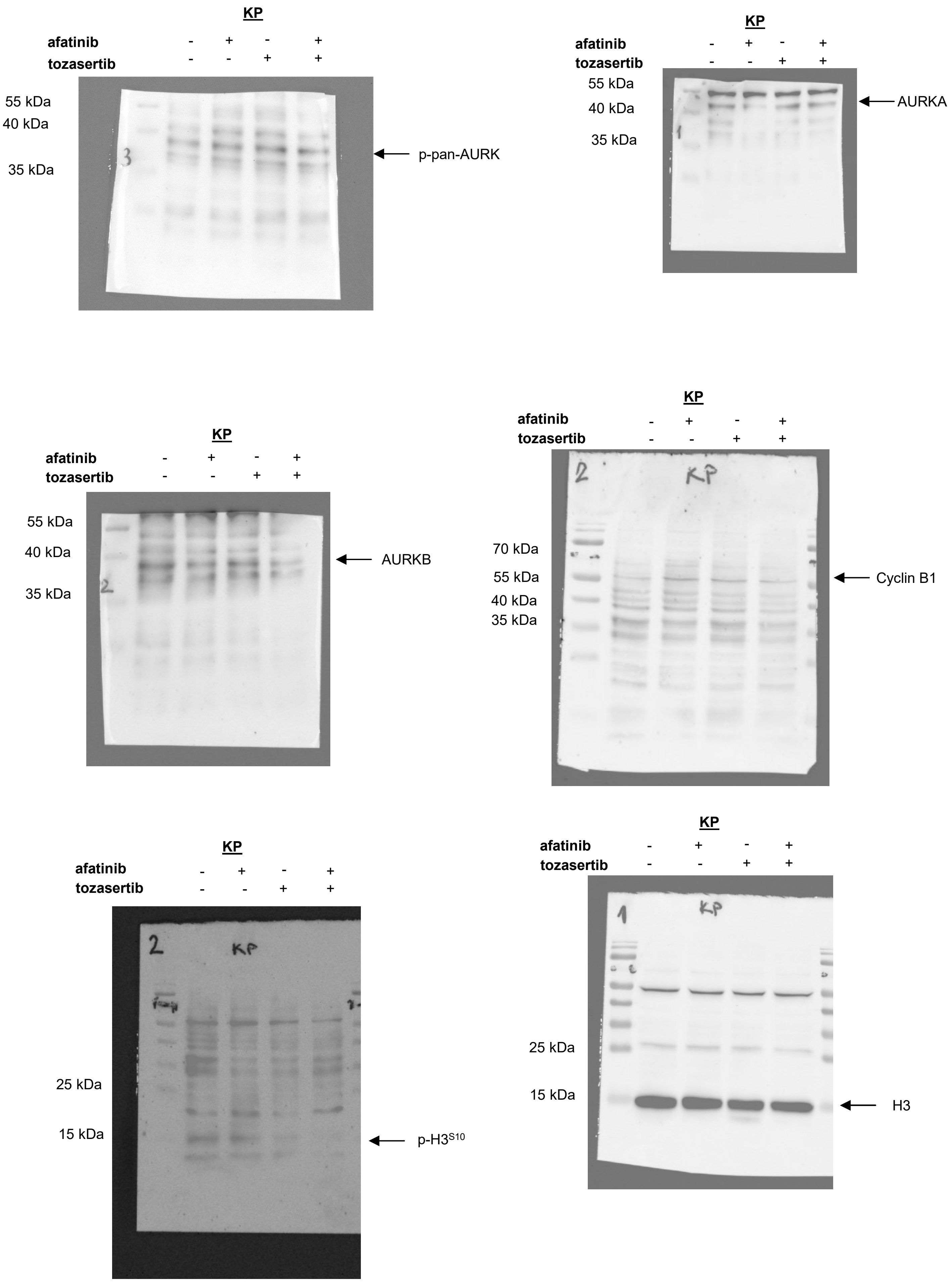

Source data for Figure 7b

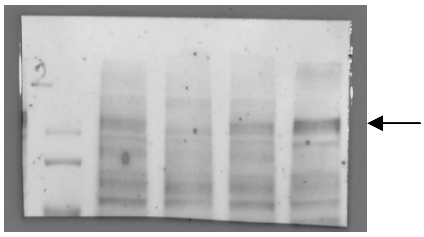

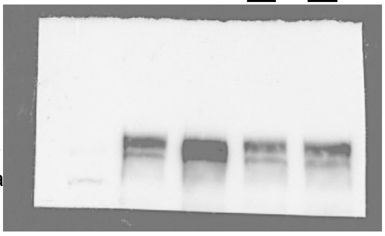

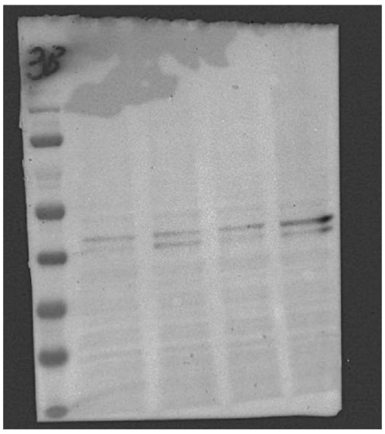

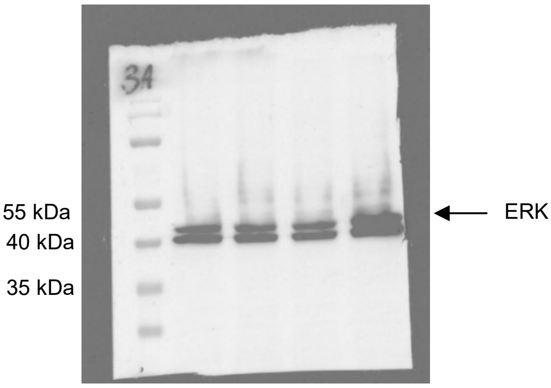

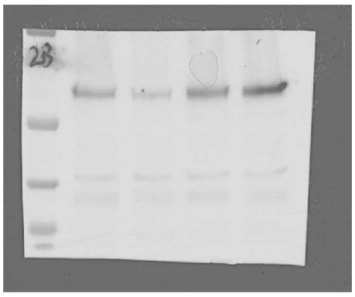

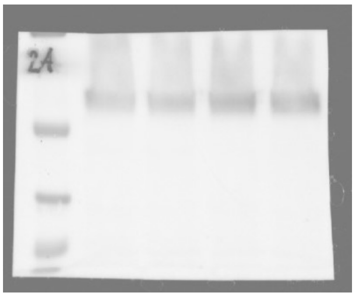

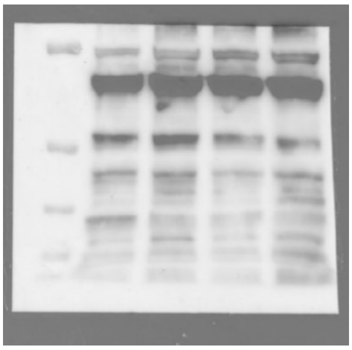

Source data for Figure 7b

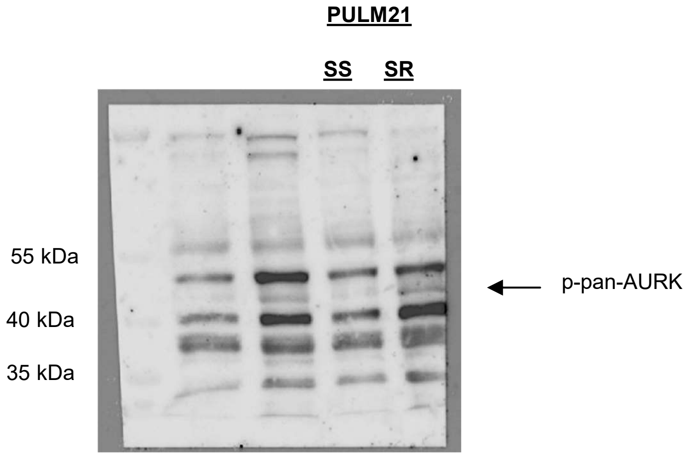

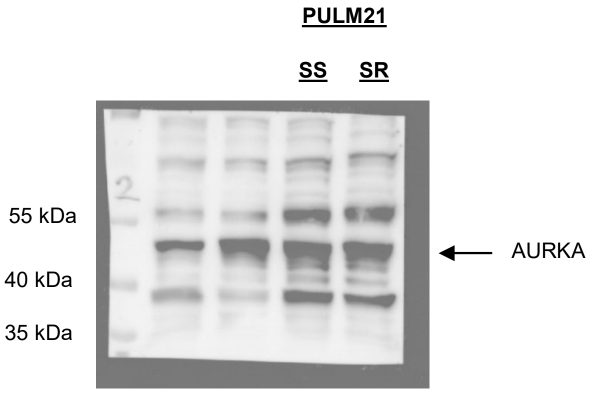

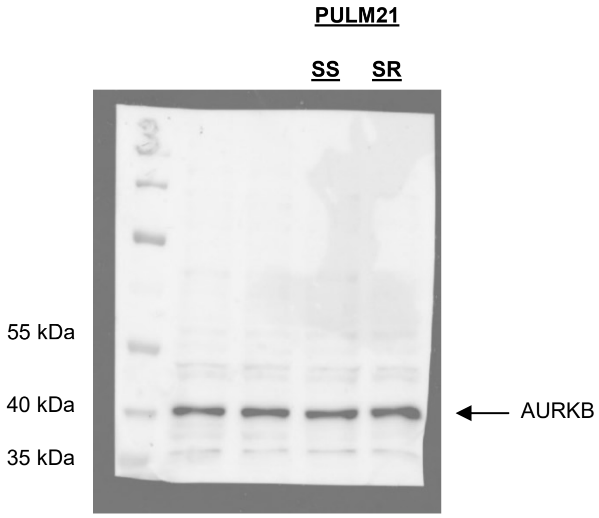

Source data for Figure 7d

PULM21-SR

-	+	-	+	afatinib
-	+	+	-	tozasertib

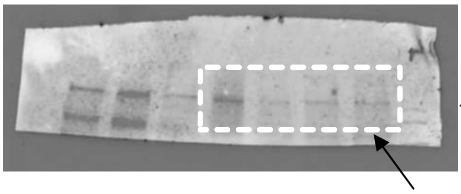

PULM21-SR

-	+	-	+	afatinib
-	+	+	-	tozasertib

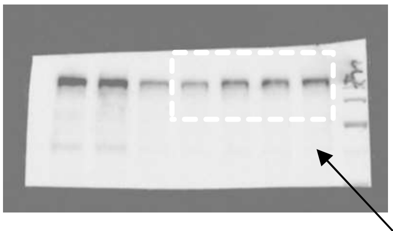

PULM21-SR

-	+	-	+	afatinib
-	+	+	-	tozasertib

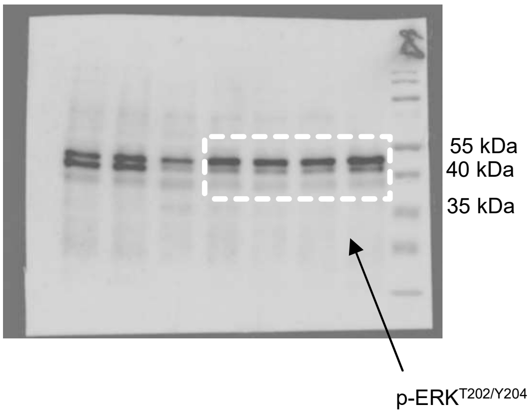

PULM21-SR

-	+	-	+	afatinib
-	+	+	-	tozasertib

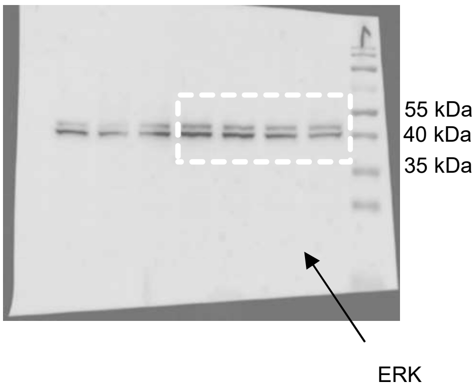

PULM21-SR

-	+	-	+	afatinib
-	+	+	-	tozasertib

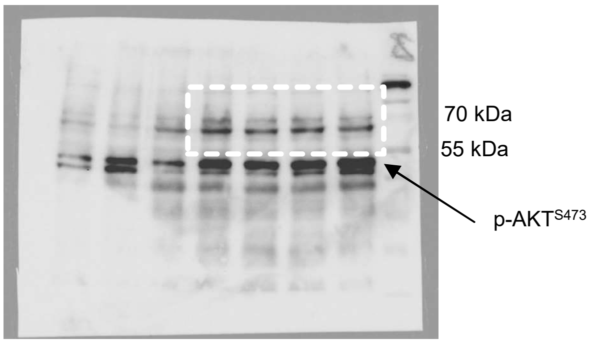

PULM21-SR

-	+	-	+	afatinib
-	+	+	-	tozasertib

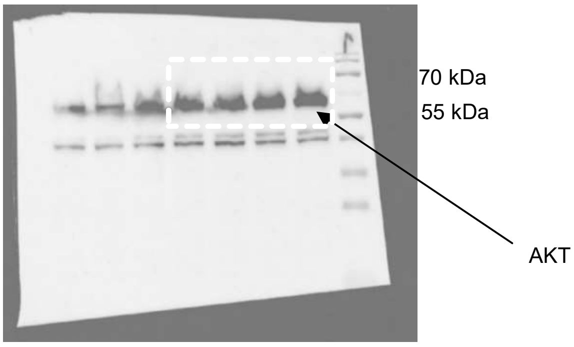

PULM21-SR

-	+	-	+	afatinib
-	+	+	-	tozasertib

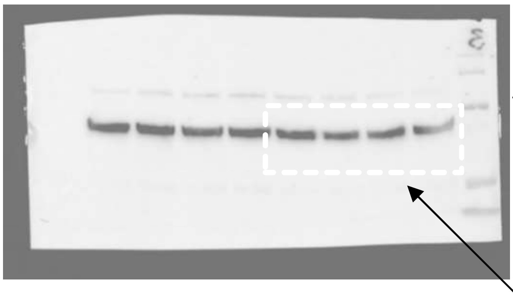

Source data for Figure 7d

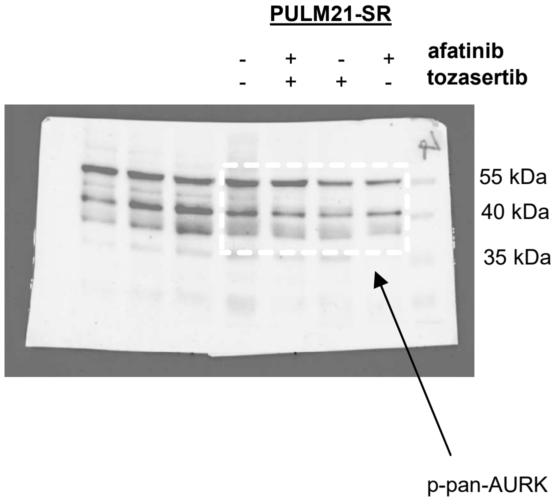

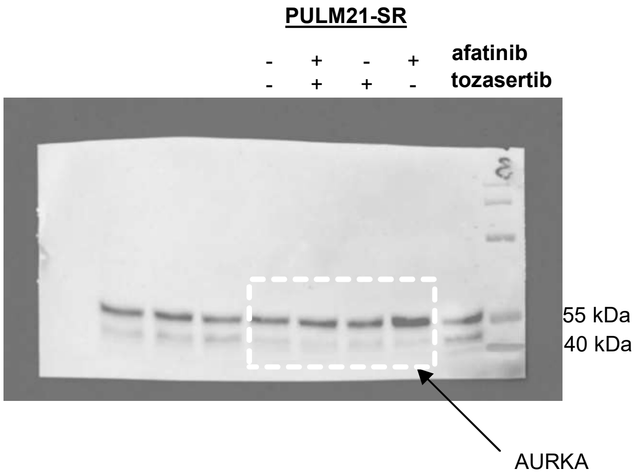

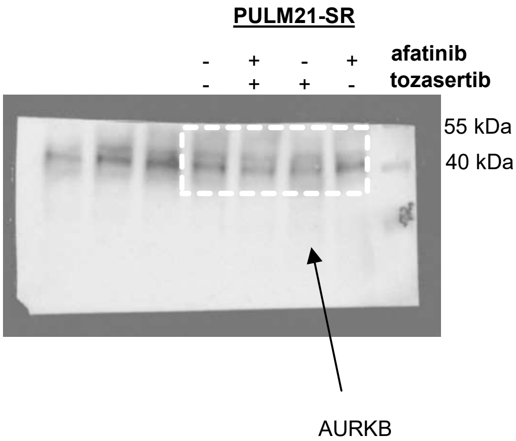

Source data for Supplementary Figure 12a

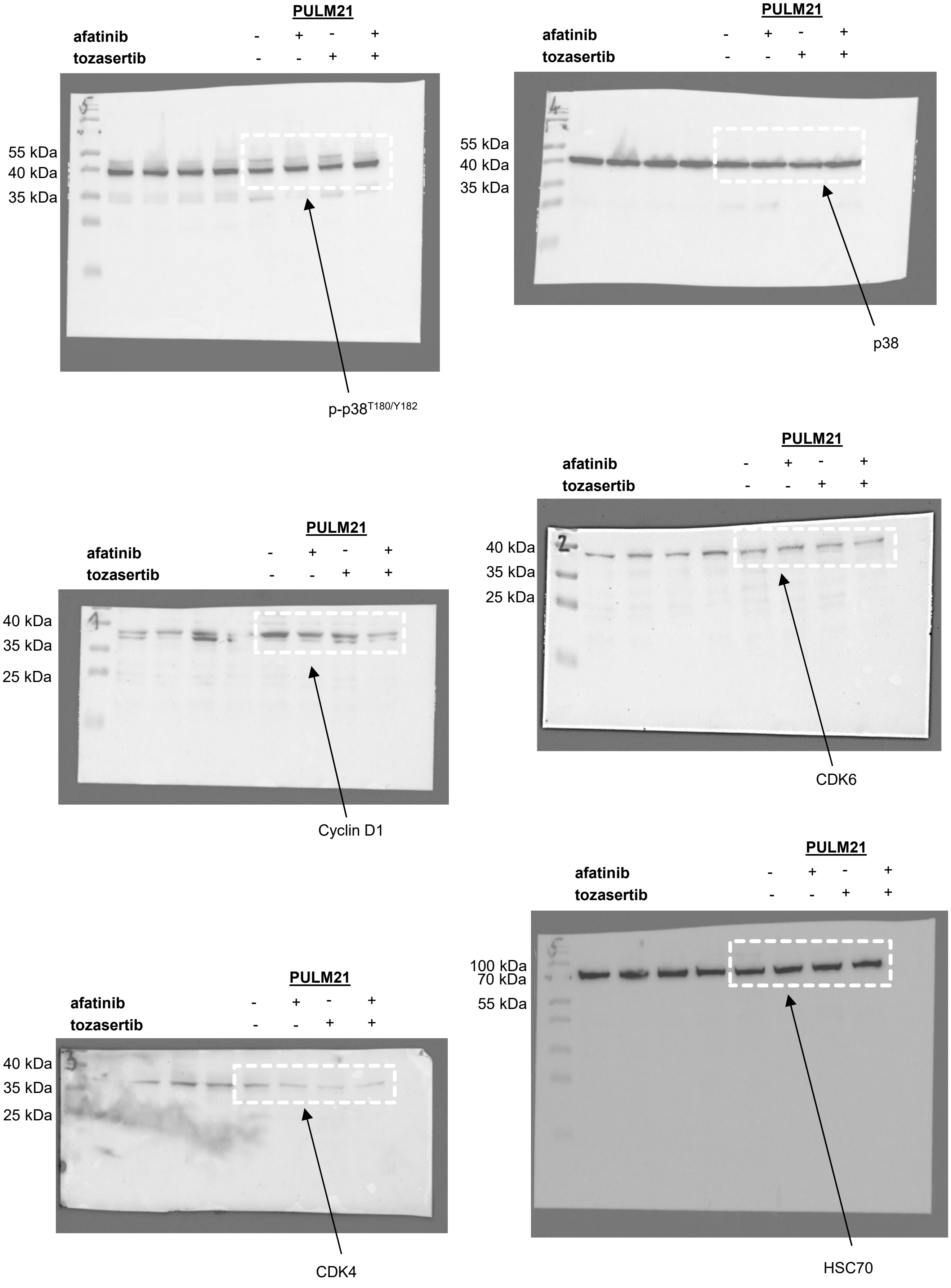

Source data for Supplementary Figure 12a

PULM21

**afatinib**	-	+	-	+
**tozasertib**	-	-	+	+

130 kDa
100 kDa
70 kDa

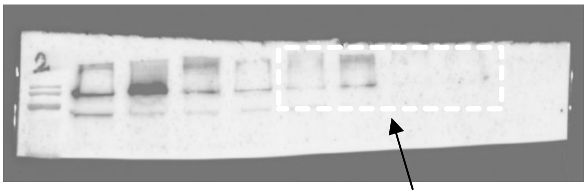

p-RB$^{S807/811}$

PULM21

**afatinib**	-	+	-	+
**tozasertib**	-	-	+	+

130 kDa
100 kDa
70 kDa

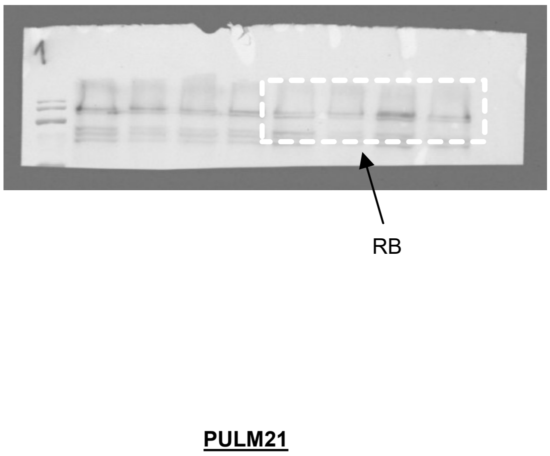

RB

PULM21

**afatinib**	-	+	-	+
**tozasertib**	-	-	+	+

100 kDa
70 kDa

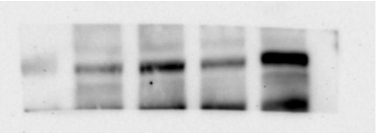

p-STAT3^{Y705}

PULM21

**afatinib**	-	+	-	+
**tozasertib**	-	-	+	+

100 kDa
70 kDa
55 kDa

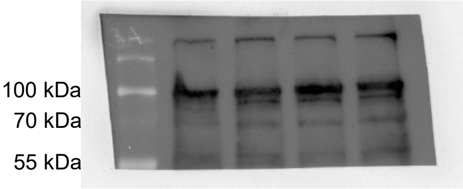

STAT3

PULM21

**afatinib**	-	+	-	+
**tozasertib**	-	-	+	+

100 kDa

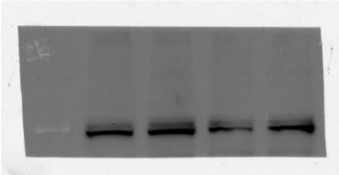

p-TBK1^{S172}

PULM21

**afatinib**	-	+	-	+
**tozasertib**	-	-	+	+

100 kDa
70 kDa
55 kDa

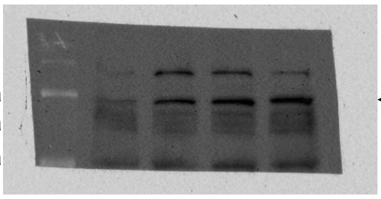

TBK1

Source data for Supplementary Figure 12b

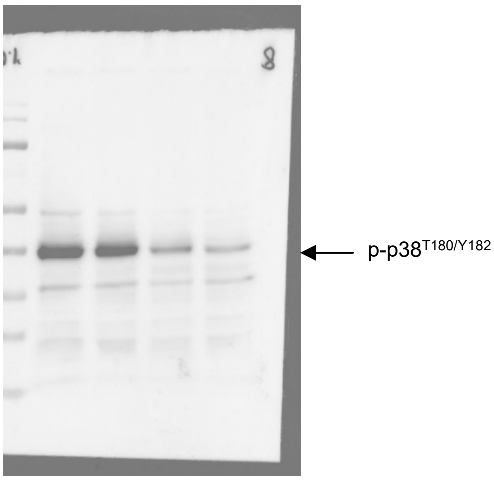

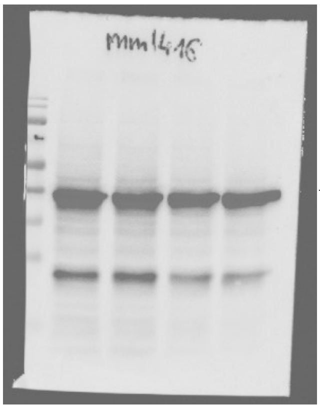

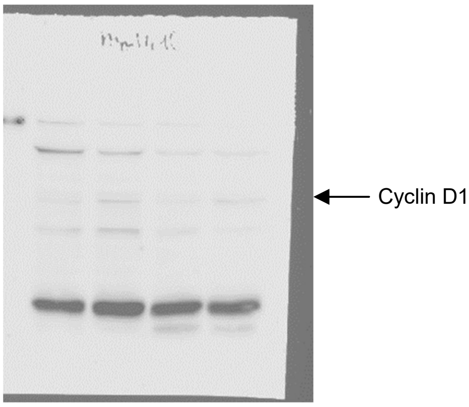

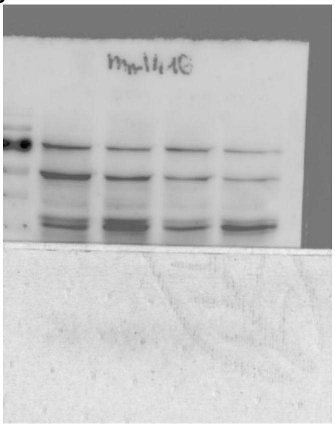

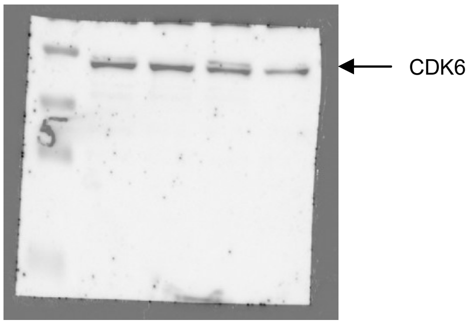

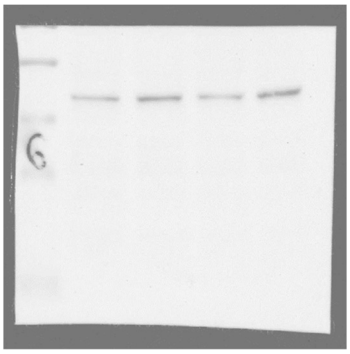

Source data for Supplementary Figure 12b

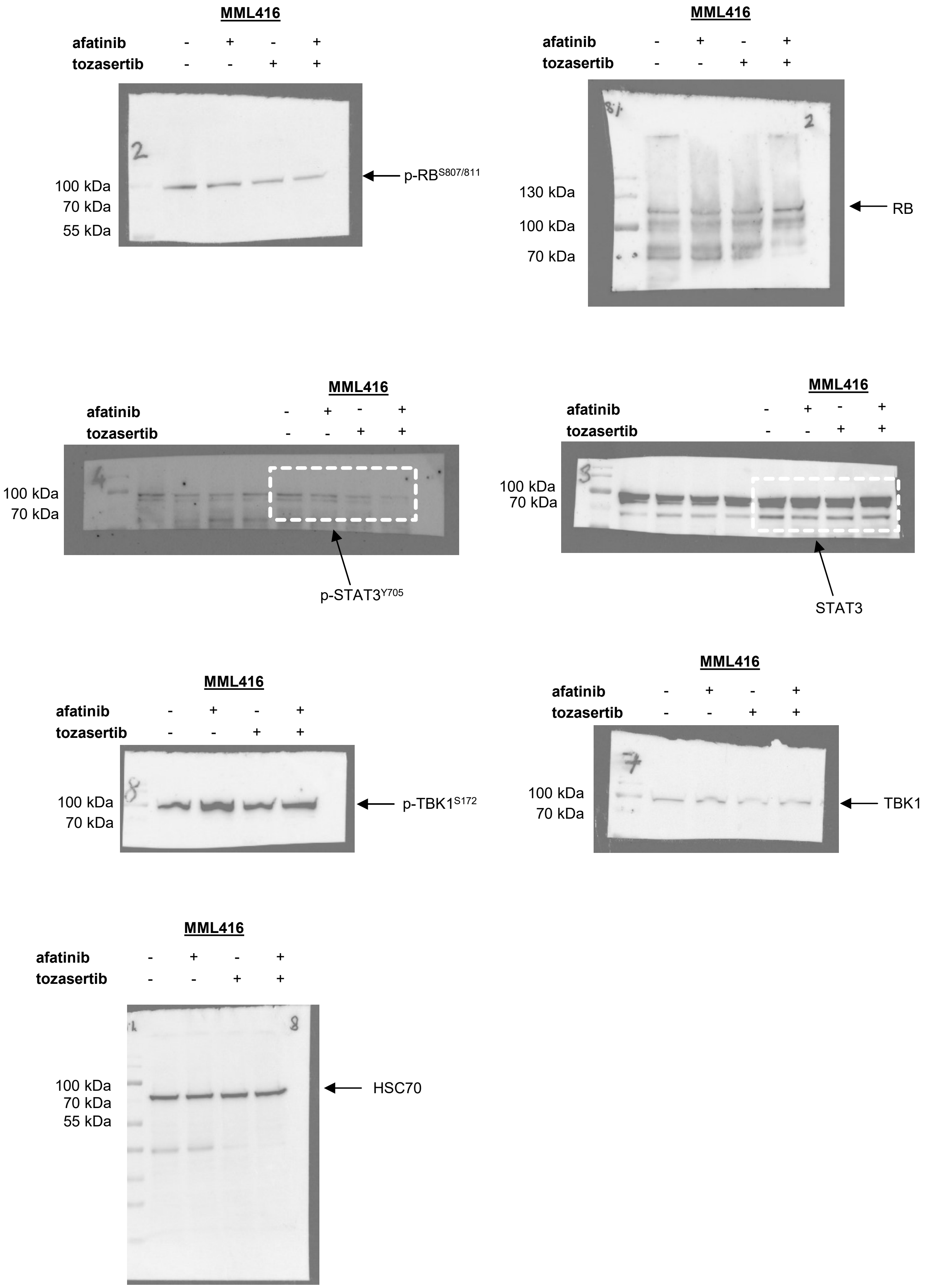

Source data for Supplementary Figure 12c

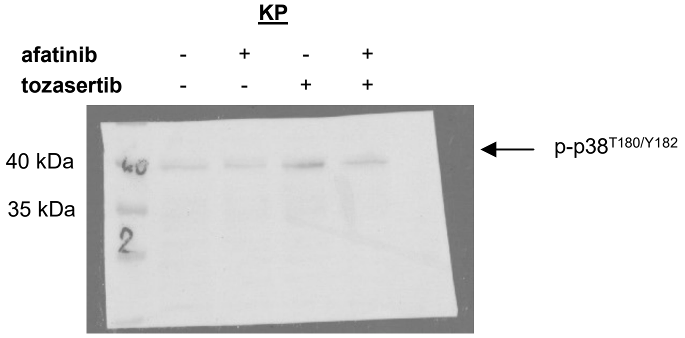

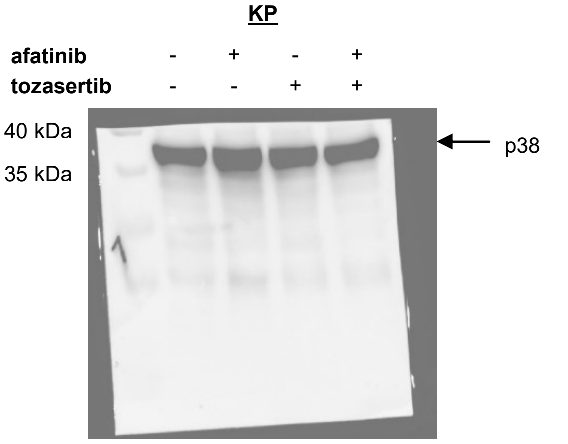

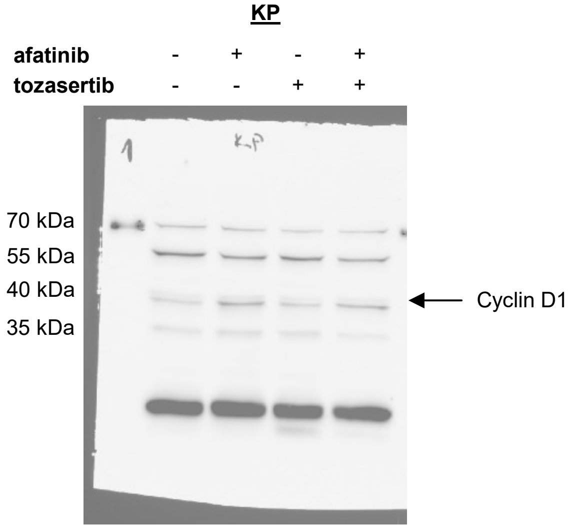

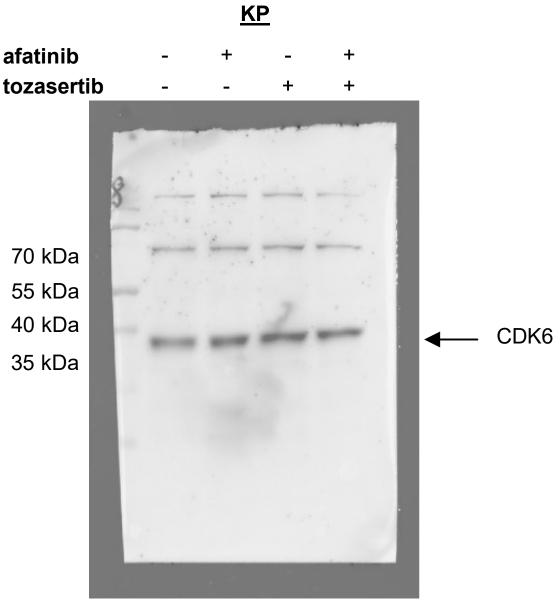

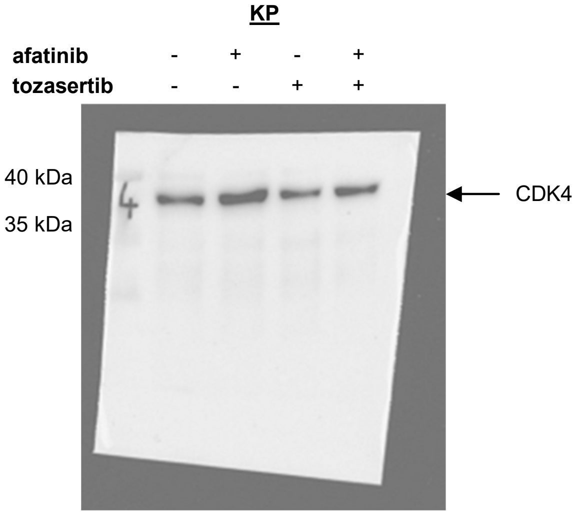

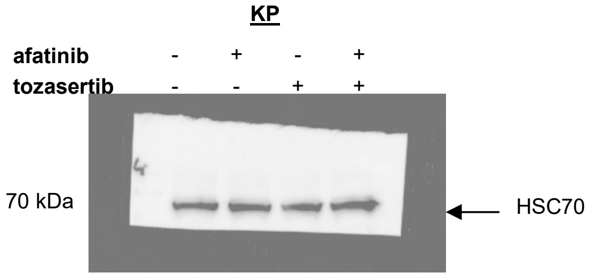

Source data for Supplementary Figure 12c

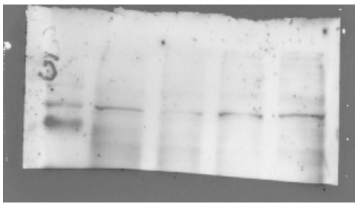

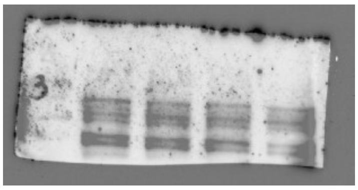

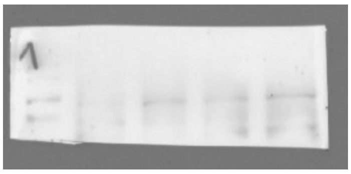

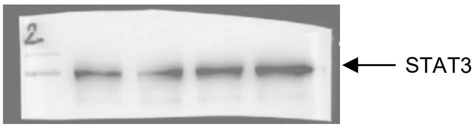

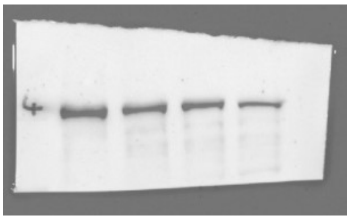

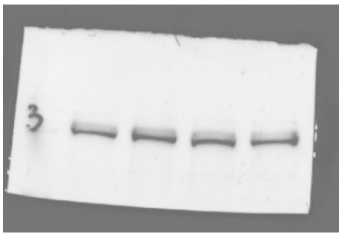

Source data for Supplementary Figure 14b

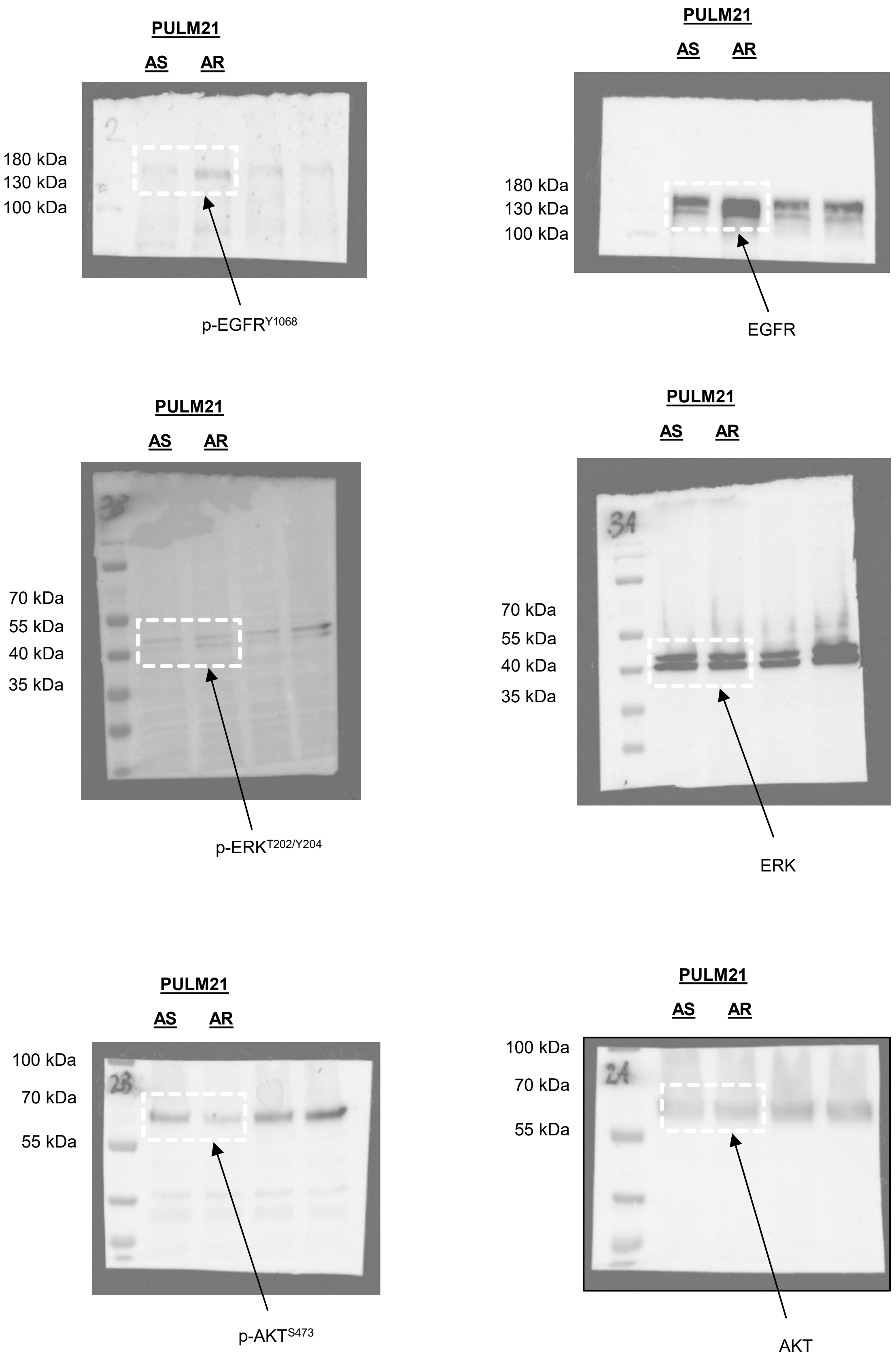

Source data for Supplementary Figure 14b

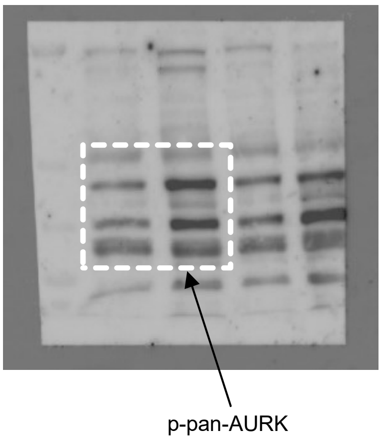

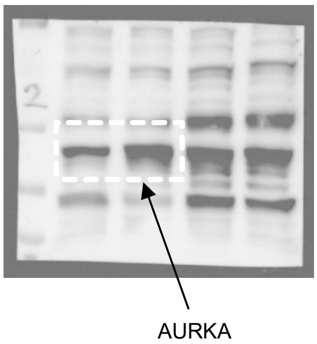

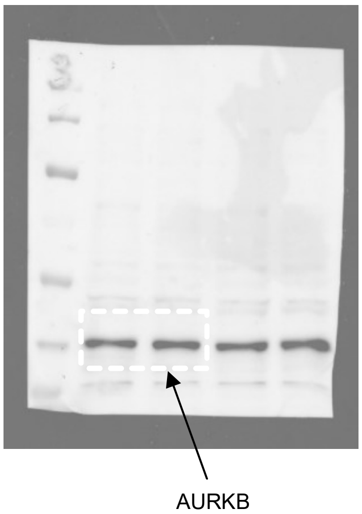

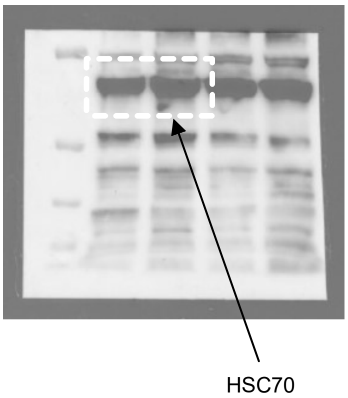

Source data for Supplementary Figure 14d

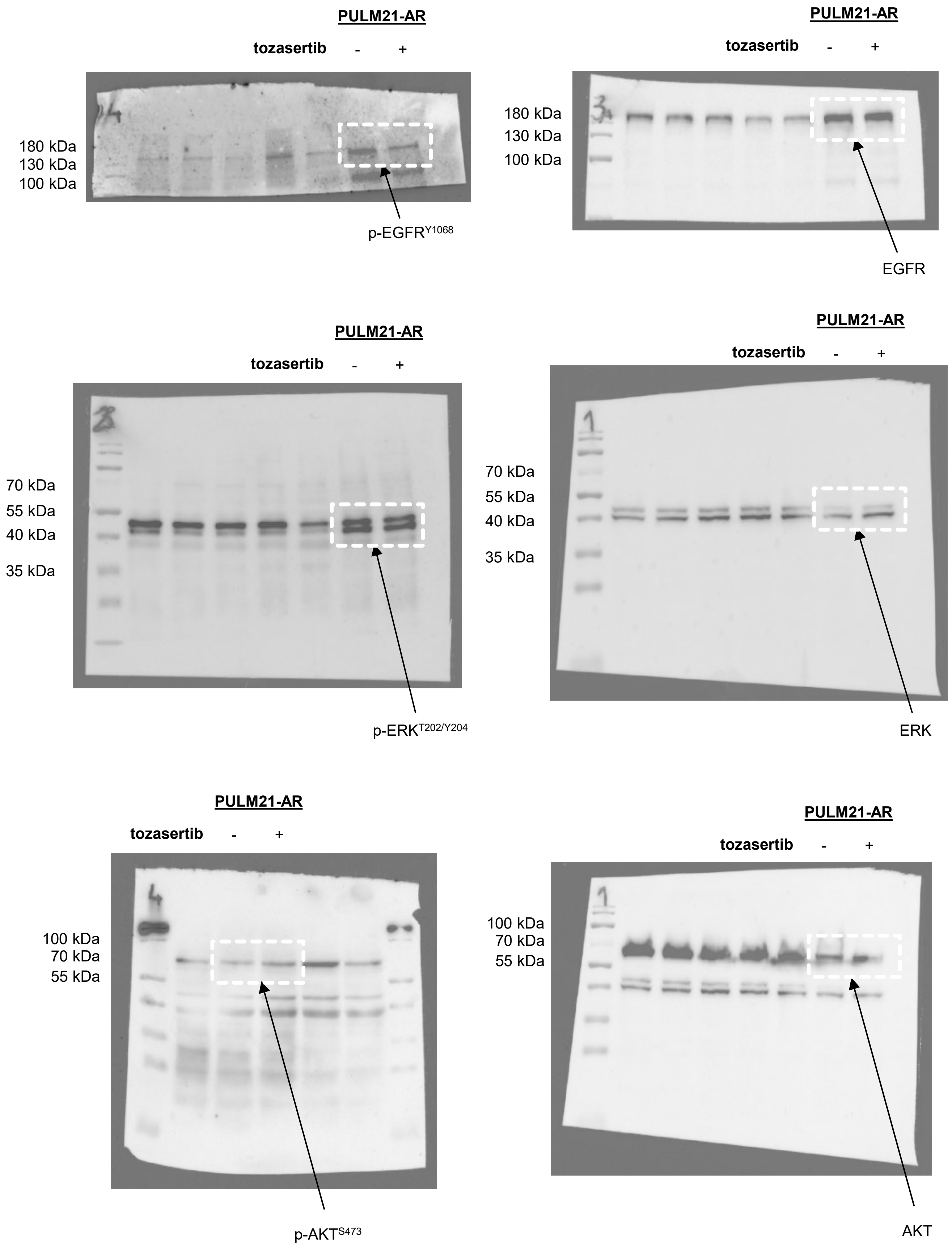

Source data for Supplementary Figure 14d

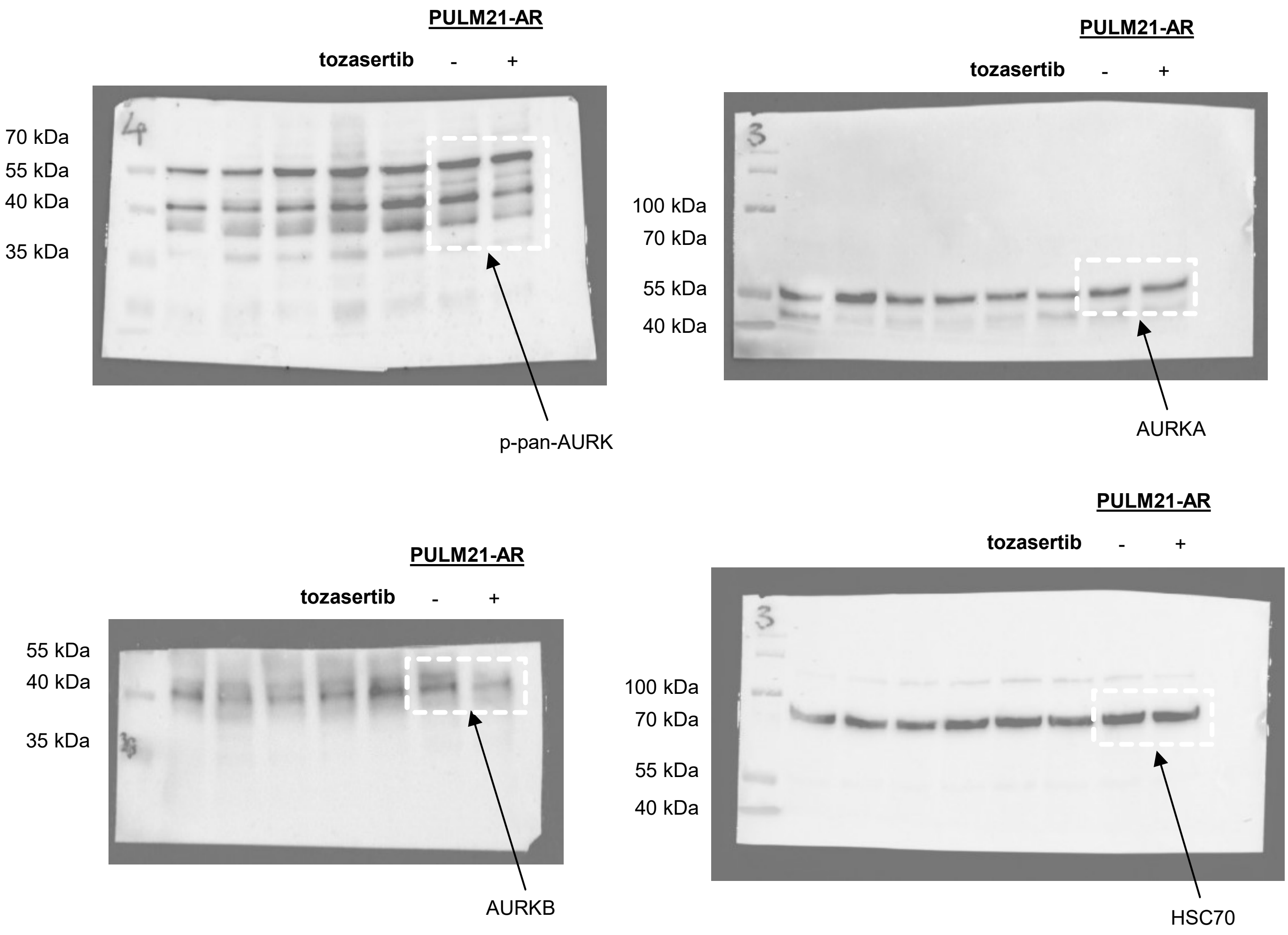

Supplement: Supplementary file 1 — revSupplementary material_IUJ [file 41698_2025_1242_MOESM1_ESM.pdf]
